# Identification of a 5-Methylcytosine Site (mC-7) That May Inhibit *CXCL11* Expression and Regulate *E. coli* F18 Susceptibility in IPEC-J2 Cells

**DOI:** 10.3390/vetsci9110600

**Published:** 2022-10-28

**Authors:** Xiaoru Shi, Luchen Yu, Rufeng Huang, Wenbin Bao, Shenglong Wu, Zhengchang Wu

**Affiliations:** 1 Key Laboratory for Animal Genetics, Breeding, Reproduction and Molecular Design of Jiangsu Province, College of Animal Science and Technology, Yangzhou University, Yangzhou 225009, China; 2 Joint International Research Laboratory of Agriculture & Agri-Product Safety, Yangzhou University, Yangzhou 225009, China

**Keywords:** *Escherichia coli*, pig, *CXCL11*, DNA methylation

## Abstract

**Simple Summary:**

*Enterotoxigenic E. coli* F18 induces post-weaning diarrhea in piglets, which results in severe economic damage for pig farmers. However, the mechanism of resistance to *E. coli* F18 infection in IPEC-J2 cells is still unclear. Here, we determined the regulatory mechanism of *CXCL11* expression in IPEC-J2 against infection by *E. coli* F18. Briefly, *CXCL11* expression was dramatically downregulated in IPEC-J2 cells infected with *E. coli* F18. In addition, *CXCL11* overexpression significantly reduced the interaction ability of *E. coli* F18 with IPEC-J2 cells. The methylation level of the mC-7 site of the CpG island in the promoter region of the *CXCL11* gene and mRNA expression were inversely linked. In addition, the mC-7 site is located in the transcription factor *OSR1*-binding domain, which is linked to the susceptibility of *E. coli* F18. In this study, the epigenetic regulation mechanism of *CXCL11* on resistance to *E. coli* F18 was revealed from the level of DNA methylation, and the resistance function genes were further identified, laying a foundation for the prevention and control strategies of resisting bacterial diarrhea in pigs.

**Abstract:**

The primary pathogen causing post-weaning diarrhea in piglets is *Escherichia coli* F18 (*E. coli* F18), hence it is essential to investigate the mechanism governing *E. coli* F18 resistance in native pig breeds. Based on the previous RNA-seq results of the duodenum from *E. coli* F18-resistant and -susceptible Meishan piglets, *CXCL11*, an important functional gene, was preliminarily screened. In this investigation, in order to further examine the expression regulation mechanism of *E. coli* F18 in intestinal porcine epithelial cells (IPEC-J2) against *E. coli* F18 infection, *CXCL11* gene expression on IPEC-J2 cells infected by *E. coli* F18 was detected, which was significantly downregulated (*p* < 0.01). Secondly, the overexpression on the IPEC-J2 cell line was successfully structured, and a relative quantification method of the *PILIN*, bacteria enumeration, and immunofluorescence assay indicated that the *CXCL11* overexpression significantly reduced the ability of *E. coli* F18 to interact with IPEC-J2 in vitro. The promoter region of the *CXCL11* gene was predicted to contain a CpG island (−619 ~ −380 bp) of which 13 CpG sites in the sequencing region were methylated to varying degrees, and the methylation level of one CPG site (mC-7) positively linked negatively with the expression of the *CXCL11* gene (*p* < 0.05). Meanwhile, a dual luciferase assay detected the mutation of the mC-7 site that significantly inhibited the luciferase activity of the *CXCL11* gene promoter (*p* < 0.01). Transcription factor prediction and expression verification indicated that mC-7 is located in the *OSR1*-binding domain, and that its expression level is related to *E. coli* F18 susceptibility. We speculated that methylation modification of the mC-7 site of the CpG island in the promoter region of the *CXCL11* gene might inhibit the binding of transcription factor *OSR1* with the mC-7 site, and then affect its expression level to regulate the susceptibility to *E. coli* F18.

## 1. Introduction

Enterotoxigenic *Escherichia coli* F18 is the primary causative pathogen of post-weaning diarrhea in piglets [1], which results in enormous economic losses to the pig trade industry because of high mortality and slow growth [2,3]. Numerous studies have reported that the immune barrier in the intestinal mucosa can prevent foreign pathogens from entering the body through the intestinal mucosa as an important defense against the invasion of external pathogens [3,4]. The intestinal mucosal immune system is composed of immunoglobulin A (IgA) and other immune factors. When pathogens invade, the intestinal mucosal immune system will initiate an inflammatory response and secrete inflammatory factors, which in turn stimulates the mucosal immune system to secrete immunoregulatory factors to protect against pathogens and prevent pathogen migration [5]. Previous studies have demonstrated that *E. coli* can increase the protein expression level of inflammatory factors when it infects intestinal epithelial cells [6,7,8] and reduces the expression levels of important proteins involved in nonspecific immunity in intestinal tissues [9,10], affecting the intestinal mucosal immune system defense against foreign pathogens and thus failing to carry out immune response. Therefore, it is of great significance to investigate the resistance connection between the immune regulatory factors affecting the intestinal mucosal immune system and *E. coli* F18 infection in weaned piglets and its regulatory mechanism. In previous studies, our research team confirmed *E. coli* F18-resistant and -susceptible Meishan weaned piglets [11]. Combined transcriptome analysis was performed in the duodenal tissue with the quantitative validation in tissue, and an important functional gene—*CXCL11*—was screened out. *CXCL11*, known as interferon-γ-inducible T-cell a-chemoattractant (I-TAC), is an important immunomodulator in the CXC chemokine family [12]. Chemokines are a subfamily of cytokines responsible for the transport of immune cells and the development of lymphoid tissue, and they play a vital role in guiding immune cell migration [13], which is necessary to initiate and deliver an effective immune response. Many studies have determined that *CXCL11* is the ligand with the highest affinity for receptor CXCR3, which can specifically activate Th1 cells expressing chemokine receptor CXCR3. Th1 mainly secretes IFN-γ and TNF-β, which are capable of mediating cellular immunity [14,15]. Current research on *CXCL11* is mainly focused on human tumor diseases [16,17]. However, the effect and regulatory mechanism of *E. coli* F18 resistance in the swine intestinal immune system are still unclear.

In eukaryotes, DNA methylation is a significant form of epigenetic alteration that occurs and occupies an extremely crucial position in regulating gene expression [18]. The primary procedure of DNA methylation is the conversion of cytosine at the 5′-end of CpG dinucleotide into 5′-methylcytosine under the action of DNA methyltransferase and methylated CpG-binding protein. A number of studies have shown that DNA methylation plays a crucial role in the regulation of immune system function and disease occurrence [19,20,21,22], and it can be used as a biomarker for disease diagnosis, prognosis assessment, and drug response prediction. Therefore, analyzing the regulation mechanisms of pig disease and immune response from the perspective of epigenetics can be an effective way to research pig disease resistance genetics and breeding. To explore the effect of the regulation mechanism of *CXCL11* gene expression and methylation modification on susceptibility to *E. coli* F18, the mRNA and protein expression changes of the *CXCL11* gene on the IPEC-J2 cells infected by *E. coli* F18 and those not infected by *E. coli* F18, and the IPEC-J2 cell line overexpressed *CXCL11* gene were constructed. The effects of *CXCL11* expression on *E. coli* F18 adhesion were verified by a relative quantification method, bacteria enumeration, and immunofluorescence assay. Next, bisulfite sequencing PCR (BSP) was used to determine the methylation degree of the CpG island in the promoter region of the *CXCL11* gene in both *E. coli* F18-infected IPEC-J2 cells and untreated IPEC-J2 cells, and we performed a correlation analysis to investigate the effect of the methylation modification of different CpG sites in the *CXCL11* gene promoter region on mRNA expression. Finally, point mutations, a dual luciferase assay, and overexpression were used to validate the effect of key CpG sites and transcription factors (TFs) on transcriptional activity in the *CXCL11* gene promoter region. Based on the new candidate molecule *CXCL11* identified previously, this study validated and explored the effect of the regulatory mechanism of its expression on resistance to porcine *E. coli* F18 and further deepened understanding of the regulatory mechanism underlying resistance to weaned piglet *E. coli* F18 from the level of DNA methylation modification. The purpose of this study was to investigate the epigenetic mechanism of porcine *CXCL11* gene expression and methylation modification on the susceptibility of *E. coli* F18 from the perspective of epigenetics, and to establish a foundation for the breeding of pigs with disease resistance.

## 2. Materials and Methods

### 2.1. Experimental Samples

The University of Pennsylvania in the USA contributed Intestinal porcine epithelial cells (IPEC-J2). *E. coli* F18ab {107/86 (O139:K12:H1)} and *E. coli* F18ac {2134P (O157:H19)} were donated by the laboratory of livestock and poultry pathogenic microorganisms, College of Veterinary Medicine, Yangzhou University. Human embryonic kidney cells (293T) were purchased from Guangzhou Jennio Biotech Co., Ltd., Guangzhou, China.

### 2.2. E. coli F18 Infection

IPEC-J2 cells were plated onto 12-well plates at a density of 5.0 × 10^5^ cells per well, and cells were cultured under the culture conditions of 5% CO2 in a 37 °C constant temperature incubator with DMEM containing 10% fetal bovine serum (FBS, GIBCO, Australia) until the cell density achieved about 80%. *E. coli* F18 cells were added to LB and incubated for 12 h on a shaking table while being a rotated at a velocity of 200 rev/min. The thalli of *E. coli* F18 were centrifuged for 10 min and gathered at a velocity of 4000 rev/min. The bacterium precipitate was resuspended in PBS, then washed three times and centrifuged. Each culture hole received 1.0 mL of bacteria that had been diluted with DMEM to 1.0 × 10^9^ CFU/mL. The cells were then harvested after being cultured for 4 h at 37 °C and 5% CO_2._

### 2.3. RNA Isolation, cDNA Synthesis and qPCR Analysis

Using the Trizol Reagent (Vazyme, Nanjing, China), the total RNA of both the *E. coli* F18-infected and -uninfected IPEC-J2 cells was extracted. Using a NanoDrop 2000 spectrophotometer (Thermo Scientific, CA, USA), agarose gel electrophoresis in formaldehyde (1%) was used to measure the whole RNA’s thickness and purity. The synthesis of cDNA was divided into two steps. The first step reaction occurred via 4 × gDNA wiper Mix 4 µL, RNA 1000 ng, and RNase free ddH_2_O up to 16 µL. Reaction conditions: 42 °C for 2 min. The second reaction system: the first reaction system was added to 5 × HiScript III qRT SuperMix 4 µL, and the total system was 20 µL. The reaction qualification was set at 85 °C for 15 min, 37 °C for 30 s, and 4 °C for preservation. The 10 µL reaction mixtures used for the qPCR assay contain 1 µL cDNA, 0.2 µL of each forward and reverse primer (10 µmol L^−1^), 5 µL 2 × AceQ Universal SYBR qPCR Master Mix, and RNase free ddH2O up to 10 µL. To guarantee the reproducibility of the results, each sample was tested in duplicate in three separate trials. An internal reference was *GAPDH* gene. All primers were synthesized by Sangon Biotech Co., Ltd., Shanghai, China (Table 1).

### 2.4. Western Blot Analysis

Proteins were extracted on the basis of instructions after IPEC-J2 cells were collected, and then they were quantified and isolated through 10% SDS-PAGE (Epizyme, Shanghai, China). PVDF membranes (Millipore, Shanghai, China) were used to receive the separated protein bands. The 5% skim milk powder was used to block the membrane, and then incubated with primary antibodies against *CXCL11* (1:1000) (Affinity Biosciences LTD) and *GAPDH* (1:5000) (Abcam, Shanghai, China) overnight at 4 °C on a shaking table, followed by the isolated protein being incubated for 2 h with secondary goat anti-rabbit or goat anti-mouse antibodies (1:500 dilution) (Proteintech Group, Wuhan, China). Finally, the proteins were made visible with an enhanced chemiluminescence (ECL) detection technique (Vazyme, Nanjing, China). The *GAPDH* protein was used as a reference.

### 2.5. Construction of CXCL11 Overexpression IPEC-J2 Cell Line

Porcine *CXCL11* gene sequences of CDS were confirmed by the NCBI. The complete CDS sequence of the porcine *CXCL11* gene was synthesized by Wuhan GeneCreate Biological Engineering Co., Ltd. (GeneCreate, Wuhan, China), and connected to the PdoubleEX-EGFP-1170593 empty vector (*CXCL11*-OE); the PdoubleEX-EGFP-1170593 empty vector was set as a negative control group (NC-OE). JetPRIME Transfection Reagent was used to transfect *CXCL11*-OE and NC-OE vectors into IPEC-J2 cells cultured once cells density reached 80%. The normal cells without transfection were used as the control group. Cells were cultured in an incubator for 4 h before replacing the original medium with fresh medium. Cells were cultured overnight to monitor the expression of green fluorescent protein (GFP). G418 100 µg/mL was used to apply the drug sieve. After two weeks of G418 selection, when all living cells were G418 resistant, RNA and protein were extracted, and *GAPDH* was used as reference to verify *CXCL11* overexpression efficiency by qPCR and Western blot.

### 2.6. Adhesion Level Detection of E. coli F18 to IPEC-J2 In Vitro

*CXCL11* overexpression and control cells were added into cell culture plates at a density of 5.0 × 10^4^ cells per well, and cultured in a thermostatic incubator with DMEM containing 10% FBS until the cell density reached roughly 80%. A relative quantification method, bacteria enumeration, and immunofluorescence assay were employed to evaluate *E. coli* F18 adhesion in vitro, investigating the interaction ability of *E. coli* F18 with IPEC-J2 cells. Bacteria enumeration: The supernatant was collected after *E. coli* F18 infection, and diluted 2000 times with PBS that was coated on the plate, at 37 °C incubator for 12 h. The bacteria colonies on the plate were counted by Image J software. A relative quantification method of *PILIN* has been published [23]. Indirect immunofluorescence assay: The cells were fixed with 500 μL 4% paraformaldehyde at 4 °C for 30 min, blended with 500 μL 0.5% Triton X-100 at room temperature for 15 min, and sealed with 200 μL blocking solution BSA at 37 °C for 2 h. In the end, 200 μL primary antibody (*E. coli*, 1:20) and 200 μL red fluorescence-labeled secondary antibody (1:200) were successively incubated. Finally, the cells were investigated and recorded under an inverted fluorescent microscope in a dark environment.

### 2.7. Methylation Detection of CpG Island in Pig CXCL11 Promoter

The 2000 bp sequence of the pig *CXCL11* 5′ end upstream promoter region was downloaded from the NCBI database. Using MethPrimer software, we predicted the *CXCL11* gene promoter region CpG island. According to the kit instructions (ZYMO, Irvine, CA, USA), the genomic DNA was identified using bisulfite sequencing PCR. The MethPrimer software was employed to create the PCR primers (F: GTTTGAAATTTAAGTTTTATGAATT, R: ATAAAAAATACAACCTCTCCCTACC) based on the sequence of the sulfite transformation. The ingredients for the 50 μL PCR amplification reactions of bisulfite-treated DNA were 4 μL of DNA template, 25 μL of ZYMO Taq Premix, 4 μL each of forward primer (10 μ M/L) and reverse primer (10 μ M/L), and 13 μL of distilled water. The purified products (230 bp) were connected to the pMD19-T vector (Takara, Dalian, China), and overnight connection at 16 °C, then transformation to DH-5α competent cells for cultivation. The transformation system contained 4.3 μL of the PCR purified product, 0.7 μL pMD-19T, and 5.0 μL solution I. All samples were sequenced using 20 positive clones in total (Invitrogen Biotechnology, Shanghai, China), and the methylation degree of sequence data was assessed using QUMA (http://quma.cdb.riken.jp/ (accessed on 4 January 2022)) software.

### 2.8. Detection of Double Luciferase Activity

A segment of the mC-7 site mutation of the *CXCL11* promoter region CpG island was connected to the pGL3-Basic vector to construct a mutant-type vector (*CXCL11*-MUT). The original sequence without mutation at the mC-7 site was linked to the pGL3-Basic vector to construct a wild-type vector (*CXCL11*-WT). The pGL3-Basic empty vector was set as a negative control group (Mock) at the same time, and three equal replicate experiments replicates were employed for each group. The 293T cells were cultured in 24-well plates and the culture conditions were 5% CO_2_ in a 37 °C constant temperature incubator. After growing to 80% density, 293T cells were co-transfected using JeTPRIM Transfection Reagent (Polyplus) with Mock, *CXCL11*-WT vector, *CXCL11*-MUT vector, respectively, and pRL Renilla luciferase reporter vector (Vazyme, Nanjing, China). After 48 h, the firefly luciferase activity was determined and standardized to Renilla. The operation procedure was implemented in rigorous accordance with the kit instructions. 

*CXCL11-*WT: GAACATTTCTCTATCGATAGGTACCCACCACAGAGGGAAAGATTTTATGAGTTTGGTTTTGGACATGTTGAGTTTGGGATTAAAAGGCACTCAAATGGGGATATTGAGTAGACAGTTTATATACAGGTCTAGAACTCAGAGAAGAAGTCTAGATTAGAAATATAATTTGGGGAGTTCCCGTCGTGGCGCAGTGGTTAACGAATCCGACTAGGAACCATGAGGTTGCAGGTTCGCTCCCTGCCCTTGCTCAGTGGGTTAAAGGATCCGGCGTTGCCGTGAGCTGTGGTGTAGGTTGCAGACGCGGCTTGGATCCC***GCGTTACTGTGGCT***CTGGCGTAGGCCAGTGGCTACAGCTCCGATTCGACCCCTAGCCTGGGAACCTCCATATGCCGCGGGAGCGGCCCAAAGAAATCTCGAGATCTGCGATCTAAGTAAG

*CXCL11-*MUT: GAACATTTCTCTATCGATAGGTACCATGCACCACAGAGGGAAAGATTTTATGAGTTTGGTTTTGGACATGTTGAGTTTGGGATTAAAAGGCACTCAAATGGGGATATTGAGTAGACAGTTTATATACAGGTCTAGAACTCAGAGAAGAAGTCTAGATTAGAAATATAATTTGGGGAGTTCCCGTCGTGGCGCAGTGGTTAACGAATCCGACTAGGAACCATGAGGTTGCAGGTTCGCTCCCTGCCCTTGCTCAGTGGGTTAAAGGATCCGGCGTTGCCGTGAGCTGTGGTGTAGGTTGCAGACGCGGCTTGGATCCC***CTTCCTTAACTTAC***CTGGCGTAGGCCAGTGGCTACAGCTCCGATTCGACCCCTAGCCTGGGAACCTCCATATGCCGCGGGAGCGGCCCAAAGAAATCTCGAGATCTGCGATCTAAGTAAG

### 2.9. Identification of Key Transcription Factor in Pig CXCL11 Promoter Region CpG Island

JASPAR (http://jaspar.genereg.net/search?q=&collection=CORE&tax_group=vertebrates (accessed on 20 February 2022)) and Alibaba2 (http://gene-regulation.com/pub/programs/alibaba2/index.html (accessed on 20 February 2022)) were adopted to estimate transcription factor-binding sites (TFBSs) on the *CXCL11* promoter region’s sequence. Based on the CpG sites of methylation level significantly altering mRNA expression, we further screened out the key TFs, and we carried out validation investigations using a dual luciferase activity assay.

### 2.10. Statistical Analyses

SPSS 25.0 software (SPSS, Inc., Chicago, IL, USA) was performed to run statistical analyses. In three separate experiments, each sample was examined in triplicate, and the relative quantitative results were processed using the 2^−ΔΔCt^ method [24]. Data were represented as the mean ± standard deviation, and comparisons were performed using Student’s t test. *p* < 0.05 or *p* < 0.01 indicated statistical significance. 

## 3. Results

### 3.1. Expression Detection of CXCL11 in the E. coli F18-Infected IPEC-J2 Cells

**Figure 1:**
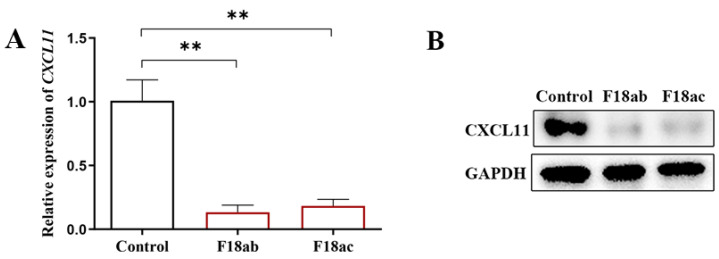
Differential expression analysis of the pig *CXCL11* gene between *E. coli* F18-infected IPEC-J2 cells and *E. coli* F18-uninfected IPEC-J2 cells. (**A**) qPCR detection, ** *p* < 0.01. (**B**) Immunoblot analysis. Original Images for Blots are in Supplementary Materials.

In order to examine the connection between *CXCL11* expression and *E. coli* F18 susceptibility in IPEC-J2, we investigated the mRNA and protein level of *CXCL11* in the *E. coli* F18-infected IPEC-J2 cells and the normal IPEC-J2 cells without *E. coli* F18 infection. As shown in Figure 1A, the expression level of *CXCL11* in the normal group was found to be substantially greater (*p* < 0.01) than that in the infection group by qPCR assays. Immunoblotting was used to further demonstrate the results, which showed that the protein levels of *CXCL11* was considerably elevated in the normal group, as shown in Figure 1B.

### 3.2. Effect of CXCL11 Overexpression on the Adhesion Ability of E. coli F18 to IPEC-J2

**Figure 2:**
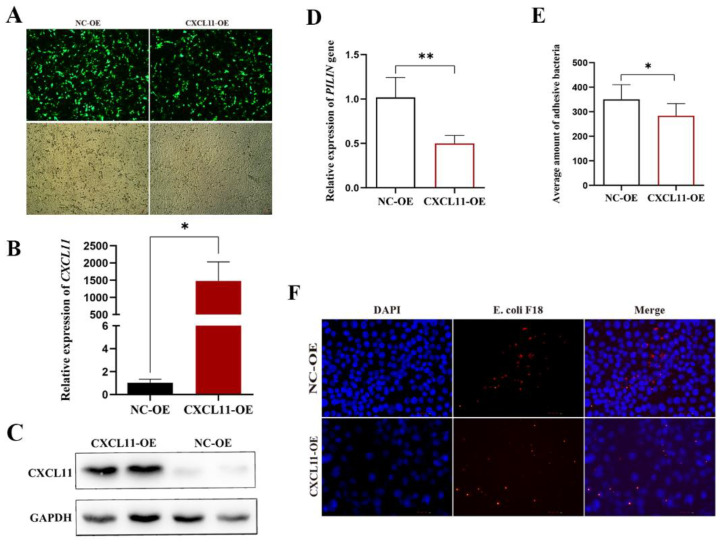
Overexpression of *CXCL11* improves *E. coli* F18 resistance. (**A**) The expression of GFP. (**B**) The mRNA expression level of the *CXCL11* gene; ** *p* < 0.01 and * *p* < 0.05. (**C**) The protein expression level of the *CXCL11* gene. (**D**,**E**) The detection of the relative quantification and bacteria enumeration. (**F**) Immunofluorescence assay. Red fluorescence signifies *E. coli* antibody, while blue fluorescence denotes nuclear staining via DAPI.

To learn more about how *CXCL11* expression impacted *E. coli* F18 invasion, this study constructed a *CXCL11* overexpression cell line (*CXCL11*-OE) and a negative control cell line (NC-OE). A relative quantification method, fimbria protein gene *PILIN* of *E. coli*, bacteria enumeration, and immunofluorescence assay were used to measure the interaction ability of *E. coli* F18 with IPEC-J2 cells in vitro by *CXCL11* overexpression. As indicated in Figure 2A, more than 80% of cells treated with *CXCL11* RNA showed signs of GFP expression. The overexpression potential of *CXCL11* in overexpression *CXCL11*-treated IPEC-J2 cells was 143,151.12% (Figure 2B). The degree of protein expression indicated that the *CXCL11*-OE group was memorably stronger than the NC-OE group (Figure 2C). Consequently, we were able to successfully create the *CXCL11* overexpression cell line. We looked into how the interaction between IPEC-J2 cells and *E. coli* F18 was impacted by *CXCL11* expression. After *CXCL11* overexpression, relative quantification (Figure 2D) showed that the adhesion ability of *E. coli* F18-expressing fimbriae in the *CXCL11*-OE group were significantly decreased than in the control groups (*p* < 0.01); bacteria enumeration (Figure 2E) showed consistency with the expression of *PILIN* (*p* < 0.05). The immunofluorescence assay revealed that the presence of *E. coli* F18 was noticeably decreased in the *CXCL11*-OE group compared to the control group (Figure 2F).

### 3.3. Detection of Methylation Level of CpG Island in Promoter Region of the CXCL11 Gene and Screening of Key CpG Sites

**Figure 3:**
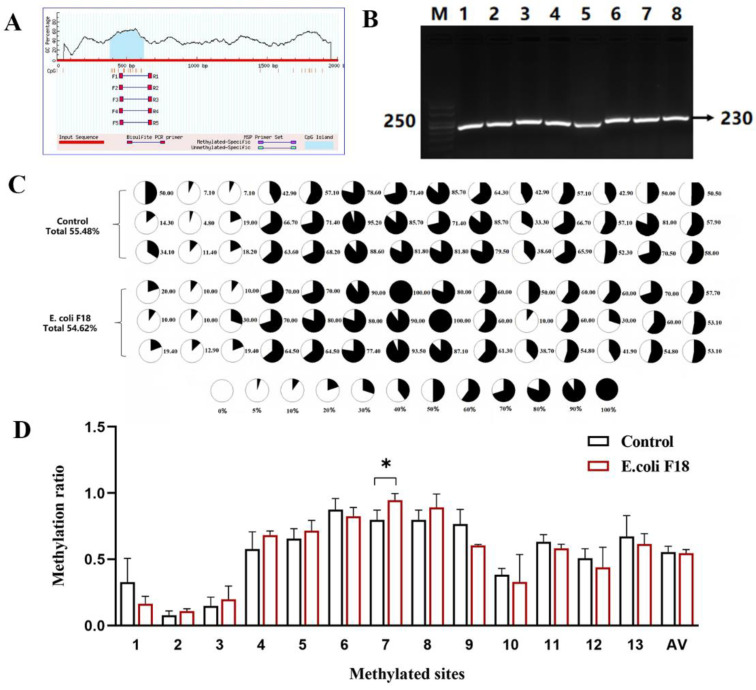
(**A**) CpG island in the *CXCL11* gene promoter region. (**B**) PCR-amplified fragment of the CpG island in the *CXCL11* gene promoter region. (**C**) The degree of methylation at the discovered CpG sites. On the x axis, 1~13 mean the different CpG sites. Pie charts are used to observe CpG sites, and the black area represents the degree of methylation. AV: the average degree of methylation. (**D**) Differential methylation analysis from *E. coli* F18-infected and -uninfected IPEC-J2, * *p* < 0.05.

MethPrimer online software was used to predict the CpG island of the *CXCL11* promoter region and found that *CXCL11* had a CpG island of 240 bp length (−619~−380 bp) (Figure 3A). We investigated the methylation degree of the *CXCL11* promoter region CpG island in *E. coli* F18-infectedand -uninfected IPEC-J2 cells via bisulfite amplicon sequencing (BSAS), and the length of the BSP-PCR amplification product was 230 bp (Figure 3B). As shown in Figure 3C, the results revealed that the 13 detected CpG sites in the CpG island of the *CXCL11* promoter region were methylated to varying degrees (Figure 3C), and one CpG (mC-7) site had significant differences between the infection and control groups (Figure 3D).

### 3.4. Effect of Key CpG Site Mutation on CXCL11 Transcription Activity

**Figure 4:**
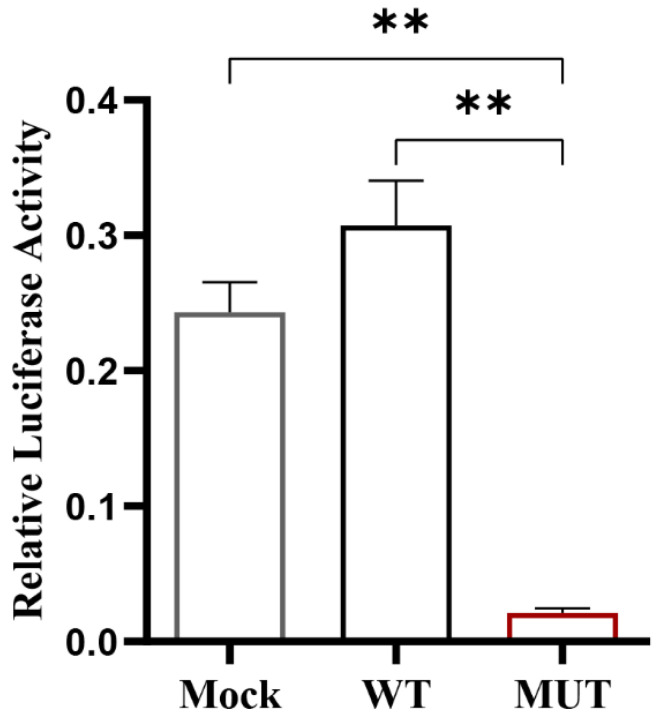
Effect of mC-7 site mutation on luciferase activity of the *CXCL11* gene promoter. Mock: empty plasmid pGL3-Basic; MUT: recombinant plasmid treated with mutation of the mC-7 site (pGL3-Basic-MUT). WT: recombinant plasmid not treated with the original sequence of the mC-7 site (pGL3-Basic-WT). The promoter sequence that was examined was present in both the MUT group and the WT group. ** *p* < 0.01.

**Figure 5:**
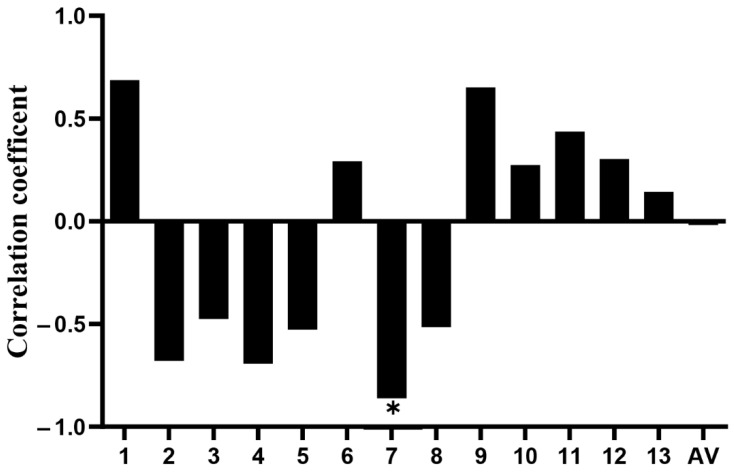
The investigation of the relationship between mRNA expression and CpG island methylation degree in the promoter region of the *CXCL11* gene, * *p* < 0.05.

Firstly, we functionally tested the effect of mutation at the key CpG site in the *CXCL11* promoter region on gene expression by luciferase assays. As shown in Figure 4, the mC-7 site mutation significantly inhibited the luciferase activity of the *CXCL11* promoter (*p* < 0.01). Secondly, the Pearson method was adopted to evaluate the connection between the methylation level of the CpG island in the *CXCL11* gene promoter region and mRNA expression. The results indicated that the correlation coefficient showed significantly negative associations between mC-7 site methylation levels and *CXCL11* mRNA expression (R = −0.862, *p* < 0.05) (Figure 5), suggesting that the high methylation of the mC-7 site probably inhibited *CXCL11* mRNA expression.

### 3.5. Evaluation of Key TFs in CpG Island of Pig CXCL11 Promoter Region

**Figure 6:**
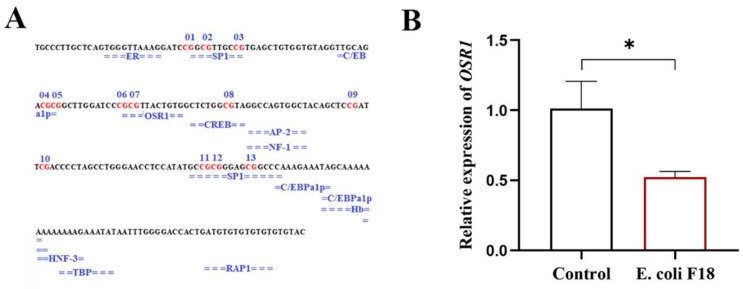
Putative TFBSs prediction results for the CpG island of the *CXCL11* promoter sequence and the relationship of the key TF with *E. coli* F18 resistance. (**A**) Putative TFBSs prediction results for the CpG island of the *CXCL11* promoter sequence. Red font indicates the methylated CpG sites, Blue font below red font means the predicted TFs. (**B**) Differential expression investigation of the key TF gene between *E. coli* F18-infected and -uninfected IPEC-J2, * *p* < 0.05.

To further authenticate the crucial TFs influencing *CXCL11* expression, according to the JASPAR and Alibaba databases, we found 14 putative TFBSs in the CpG island of the pig *CXCL11* genes’ promoter region (Figure 6A). Furthermore, mC-7 was located in the *OSR1*-binding domain, which revealed its crucial role to promoting the expression degree of *CXCL11* by affecting the interaction of TFs with promoter sequence. qPCR was employed to investigate the mRNA expression level of *OSR1* in the infection group, which was dramatically fewer than in the normal group (*p* < 0.05) (Figure 6B).

**Figure 7:**
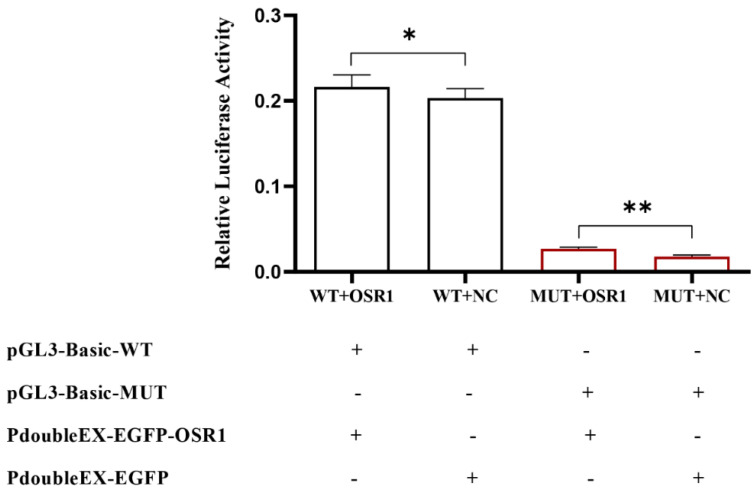
Effects of the transcription factor *OSR1* overexpression on the *CXCL11* gene promoter relative luciferase activity; * *p* < 0.05 and ** *p* < 0.01.

In order to further prove the impact of *OSR1* on *CXCL11* mRNA expression, the impact of *OSR1* overexpression on *CXCL11* gene transcriptional activity was detected by double luciferase assay in this study. The results indicated (Figure 7) that overexpression of *OSR1* significantly increased luciferin activity (*p* < 0.05).

## 4. Discussion

*E. coli* F18 is the most common adhesin pili in post-weaning bacterial diarrhea (PWD) [25,26]. Adhesins are a kind of filamentous protein expressed on the surface of the bacterial cell membrane, usually composed of the same 100 subunit structure and various minor accessory proteins, which is a crucial component of. *E. coli* F18′s pathogenicity [27]. The first step in *E. coli* F18 infection of the intestine relies on adhesins binding to the host’s intestinal epithelial cells, and the intestinal mucosal immune response is an important line of defense against pathogen invasion of the body [3,4]. Therefore, it is important to investigate the chemokine *CXCL11*, which affects the immune response of the intestinal mucosa in weaning piglets against invasion by *E. coli* F18. Based on our research, we obtained *E. coli* F18-resistant and -susceptible Meishan weaned piglets, and transcriptome analysis was performed on the duodenal tissue combined with quantitative tissue verification that screened out an important functional gene, *CXCL11*. *CXCL11*, a chemokine belonging to the CXC subfamily, is induced by IFN-c and IFN-b. The study demonstrated that chemokines play an important role in immune cell migration-mediated immune response [13]. Therefore, it was important to investigate *CXCL11*, a chemokine affecting intestinal mucosal immune response, and then to effectively resist *E. coli* F18 invasion in weaned piglets. In this investigation, qPCR and Western blot were used to detect the *CXCL11* gene expression level on the IPEC-J2 cells stimulated by different strains of *E. coli* F18 and the normal IPEC-J2 cells. The findings indicated that *CXCL11* gene expression was dramatically downregulated on IPEC-J2 cells infected by *E. coli* F18ab and *E. coli* F18ac (*p* < 0.01). In conclusion, the high expression of the *CXCL11* gene may benefit weaned piglets in their resistance to *E. coli* F18 infection. Thus, the overexpression IPEC-J2 cell line of the porcine *CXCL11* gene was constructed to explore the biological function of *CXCL11*. The detection included a relative quantification of the *PILIN*, bacteria enumeration, and immunofluorescence assay, which indicated that the *CXCL11* overexpression significantly reduced the *E. coli* F18 ability to interact with IPEC-J2 in vitro, which suggested that *CXCL11* plays a vital role in the immune regulation of *E. coli* F18 infection, and the upregulation of its expression may enhance the resistance of piglets to *E. coli* F18.

**Figure 8:**
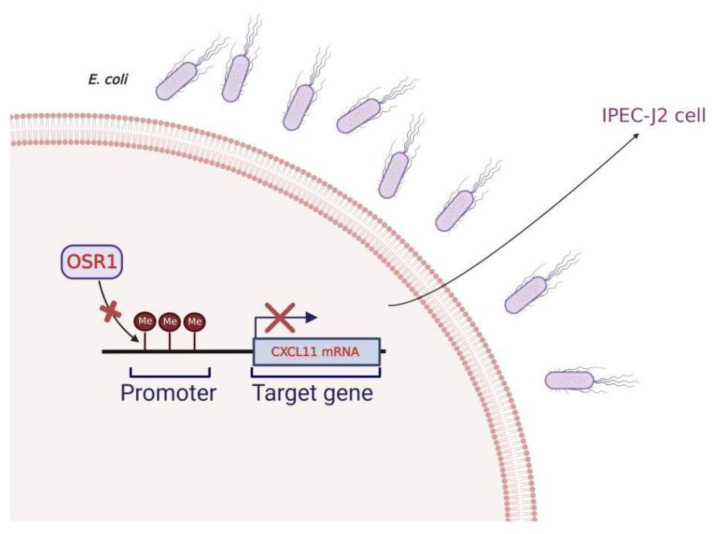
A working model summarizing the mechanism of the *CXCL11* gene in regulating *E. coli* F18 susceptibility in IPEC-J2 cells.

At present, DNA methylation is a prominent topic in epigenetics study because it regulates gene expression and has a crucial role in the genesis of illness. [28,29,30]. Almost all methylation of the vertebrate genome occurs in the cytosine of CpG dinucleotides. For example, 70–80% of CpG is methylated in the human genome [31]. In mammals, highly aggregated CpG islands exist in the transcriptional regulatory region [32,33,34]. In order to further examine the expression regulation mechanism of IPEC-J2 cells resistant to *E. coli* F18 infection at the cellular level, MethPrimer was used in this study to predict the presence of a CpG island in the *CXCL11* gene’s promoter region. Methylation sequencing revealed that the 13 CpG sites in the sequencing region were methylated to varying degrees, and one of the CpG sites (mC-7) had a significant difference in methylation level between the *E. coli* F18-infected and -uninfected IPEC-J2 cells. Studies have confirmed that the DNA methylation of FUT1 and FUT2 promoters controls their expression, which in turn controls weaned piglets’ resistance to the ETEC F18 pathogen [35,36]. Additionally, there are also studies indicating that ETEC F4ab/ac infection can trigger variation in DNA methylation and alter the expression of genes associated with immune responses [37]. Therefore, piglets infected with *E. coli* F18 may trigger significant levels of methylation at the mC-7 site in the *CXCL11* gene’s promoter region, and then show susceptibility to *E. coli* F18 in weaned piglets. In addition, the impact of the mC-7 site mutation on *CXCL11* gene promoter activity was detected by dual luciferase assay. The findings determined that the mutation of the mC-7 site significantly inhibited the luciferase activity of the *CXCL11* gene promoter (*p* < 0.01). The two above conclusions suggest that the mC-7 site may be a key regulatory site in the *CXCL11* gene’s promoter region for resistance to *E. coli* F18 infection in piglets. In our investigation, based on the fact that promoter methylation is directly related to gene transcription [33], the Pearson method was further used to analyze the relationship between the methylation levels at each CpG site in the CpG island in the promoter region of the *CXCL11* gene and mRNA expression. The results revealed that the methylation degree of the mC-7 site was inversely linked with the mRNA expression of the *CXCL11* gene (*p* < 0.05), and the mC-7 site was located in the *OSR1*-binding domain. *OSR1* is a protein composed of 266 amino acids, encoding a zinc finger transcription factor. Recently, studies have shown that *OSR1*, a novel tumor suppressor gene, is preferentially methylated in gastric cancer cell lines and its expression is reduced or silenced [38], thus it plays a role as a tumor suppressor, which is also an important regulatory factor in embryo, heart, and genitourinary development [39,40]. In many genes whose GC sequences are rich in promoter regions, the binding of TFs and the target gene promoter can be blocked by promoter methylation modification, resulting in target gene silencing [41,42]. Based on the inhibition of gene expression by DNA methylation modification in the promoter region, the transcription inhibition is exerted by hindering the recognition of TFBSs, which in turn leads to the inability of TFs to bind to promoters. This study speculated that the high methylation of the mC-7 site of the CpG island in the promoter region of the *CXCL11* gene may block the binding of *OSR1*, further inhibiting the expression of the *CXCL11* gene. The study determined that most TFs also take part in the regulation of gene expression during pathogenic infection. In order to more accurately determine whether *OSR1* was involved in the regulatory mechanism of *E. coli* F18 resistance, qPCR was adopted to detect the mRNA expression of *OSR1* on IPEC-J2 cells infected by *E. coli* F18 and IPEC-J2 cells not infected by *E. coli* F18. The findings showed that the mRNA expression level of *OSR1* in the infected group was dramatically lower than in the normal group (*p* < 0.05), which implied a connection between the degree of *OSR1* expression and the susceptibility to *E. coli* F18. Meanwhile, *OSR1* may have a role in the mechanism governing E. coli F18 resistance. In order to further verify the influence of *OSR1* on the mRNA expression of the *CXCL11* gene, the double luciferase assay was employed to investigate the influence of *OSR1* overexpression on *CXCL11* gene transcriptional activity. We found that luciferase activity was significantly increased after overexpression of *OSR1* (*p* < 0.05). The results suggest that *OSR1* may be an important transcription factor in the transcription regulation of the *CXCL11* gene. In conclusion, this study theorized that methylation modification of the mC-7 site of the CpG island in the promoter region of the *CXCL11* gene might inhibit the binding of *OSR1* with the mC-7 site and then affect its expression level to regulate the susceptibility to *E. coli* F18 (Figure 8). However, it is necessary to further study the regulation of TFs on the *CXCL11* promoter at the cellular level using an electrophoresis mobility shift assay (EMSA) method, etc., in order to offer a more complete experimental underpinning for the epigenetic regulation mechanism of the *CXCL11* gene.

## 5. Conclusions

This study verified at the cellular level that *CXCL11* expression is closely related to the resistance of IPEC-J2 to *E. coli* F18 infection. In addition, the *CXCL11* gene CpG island was identified through bioinformatics analysis. Methylation and double luciferase analysis determined that the key CpG site (mC-7) had an important effect on the activity of the *CXCL11* gene promoter region, which indicated that the high methylation of the mC-7 site may block the binding of *OSR1* and then affect its expression level to regulate the susceptibility to *E. coli* F18. This research further clarified the resistance functional genes, identified the epigenetic regulatory mechanism of *CXCL11* resistance to *E. coli* F18 from the level of DNA methylation, deepened our understanding of the regulatory mechanism of *E. coli* F18 resistance in weaned piglets from the standpoint of DNA methylation modification, and established a foundation for the future development of prevention and control measures as well as breeding for disease resistance of porcine bacterial diarrhea.

## Figures and Tables

**Table 1:** The qPCR primers information.

Gene Name	GenBank Accession No.	Primer Sequence	Fragment Size (bp)
*CXCL11*	XM_005666774.3	F:5′- TCAAAGCGGGAAGGTGTCTT -3′	301
		R:5′- TGCTTTCAGGGTGACAATCACTT -3′	
*OSR1*	XM_021087818.1	F:5′- GGTGGAGAGGGTGTTTCAGG-3′	166
		R:5′- CTCCACCATCCAGCTCCCAG-3′	
*GAPDH*	AF017079.1	F: 5′-ACATCATCCCTGCTTCTACTGG-3′	188
		R: 5′-CTCGGACGCCTGCTTCAC-3′	
*PILIN*	M25302.1	F: 5′-AGGCCGAACCAAAGAAGCAT-3′	117
		R: 5′-TCACCATCAGGGTTTCTGAGT-3′	
*β-actin*	NC_010445.3	F: 5′-GTCGTACTCCTGCTTGCTGAT-3′	119
		R: 5′-CCTTCTCCTTCCAGATCATCGC-3′	

## Data Availability

The datasets generated during the current study are available from the corresponding author upon reasonable request.

## Supplementary Materials

The following supporting information can be downloaded at: https://www.mdpi.com/article/10.3390/vetsci9110600/s1, original images for blots.
