# Supplementary figures and images for: Identification of a 5-Methylcytosine Site (mC-7) That May Inhibit CXCL11 Expression and Regulate E. coli F18 Susceptibility in IPEC-J2 Cells
Source: Vet Sci. 2022 Oct 28;9(11):600. doi: 10.3390/vetsci9110600 (PMC9698616; doi:10.3390/vetsci9110600)

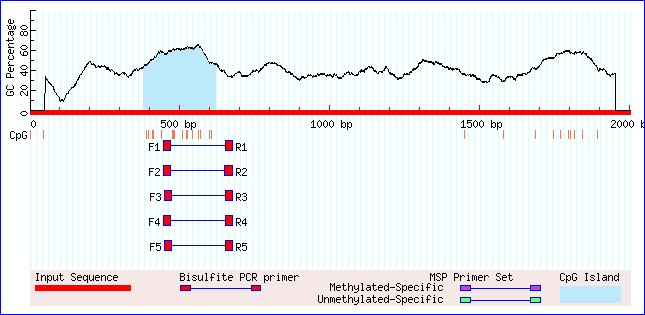

Supplement: Supplementary file 1 [file vetsci-09-00600-s001.zip › vetsci-1934507-supplementary/original source points/Methylation/CpG Island.tif]

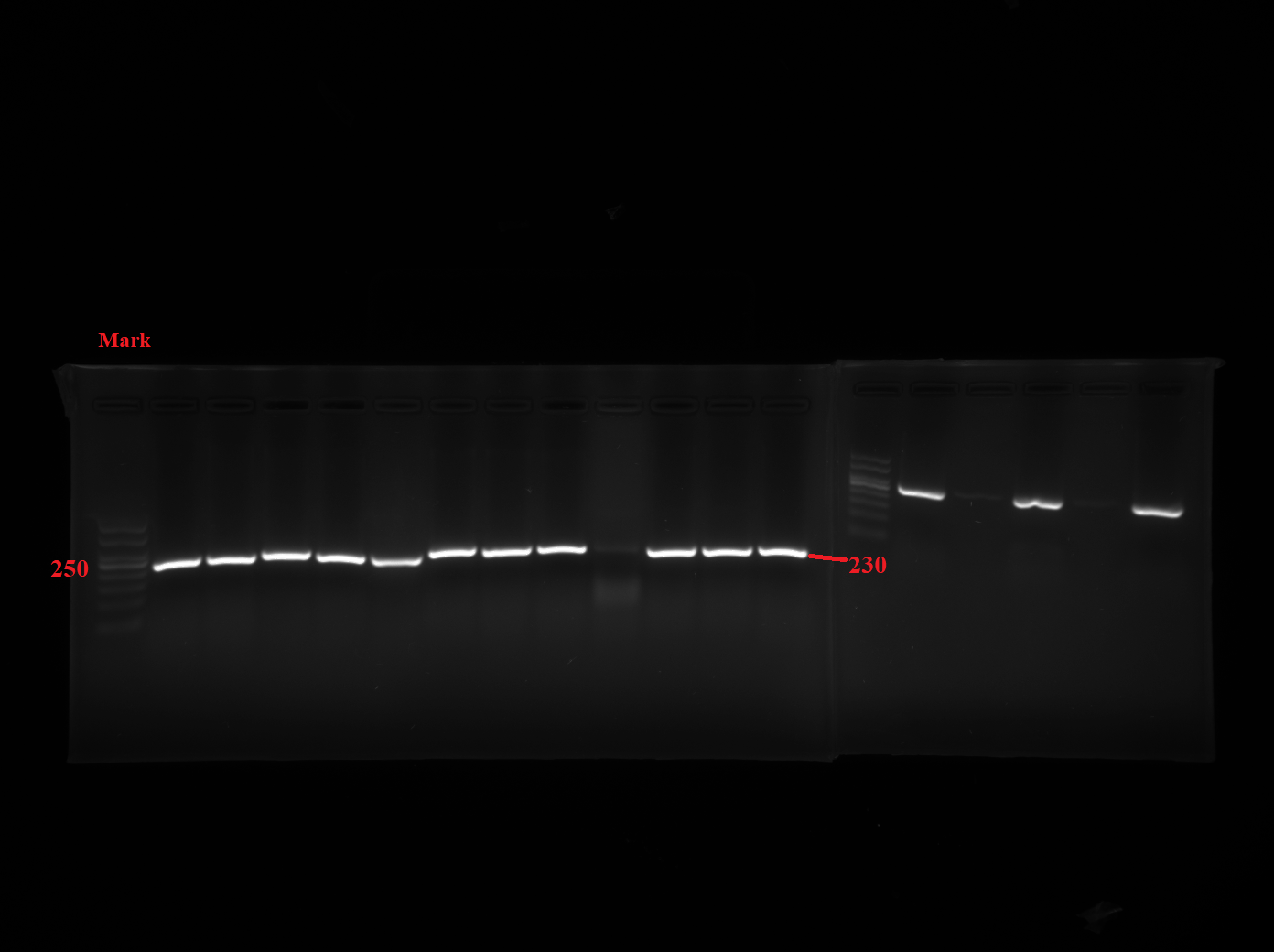

Supplement: Supplementary file 1 [file vetsci-09-00600-s001.zip › vetsci-1934507-supplementary/original source points/Methylation/Methylation PCR products-.tif]

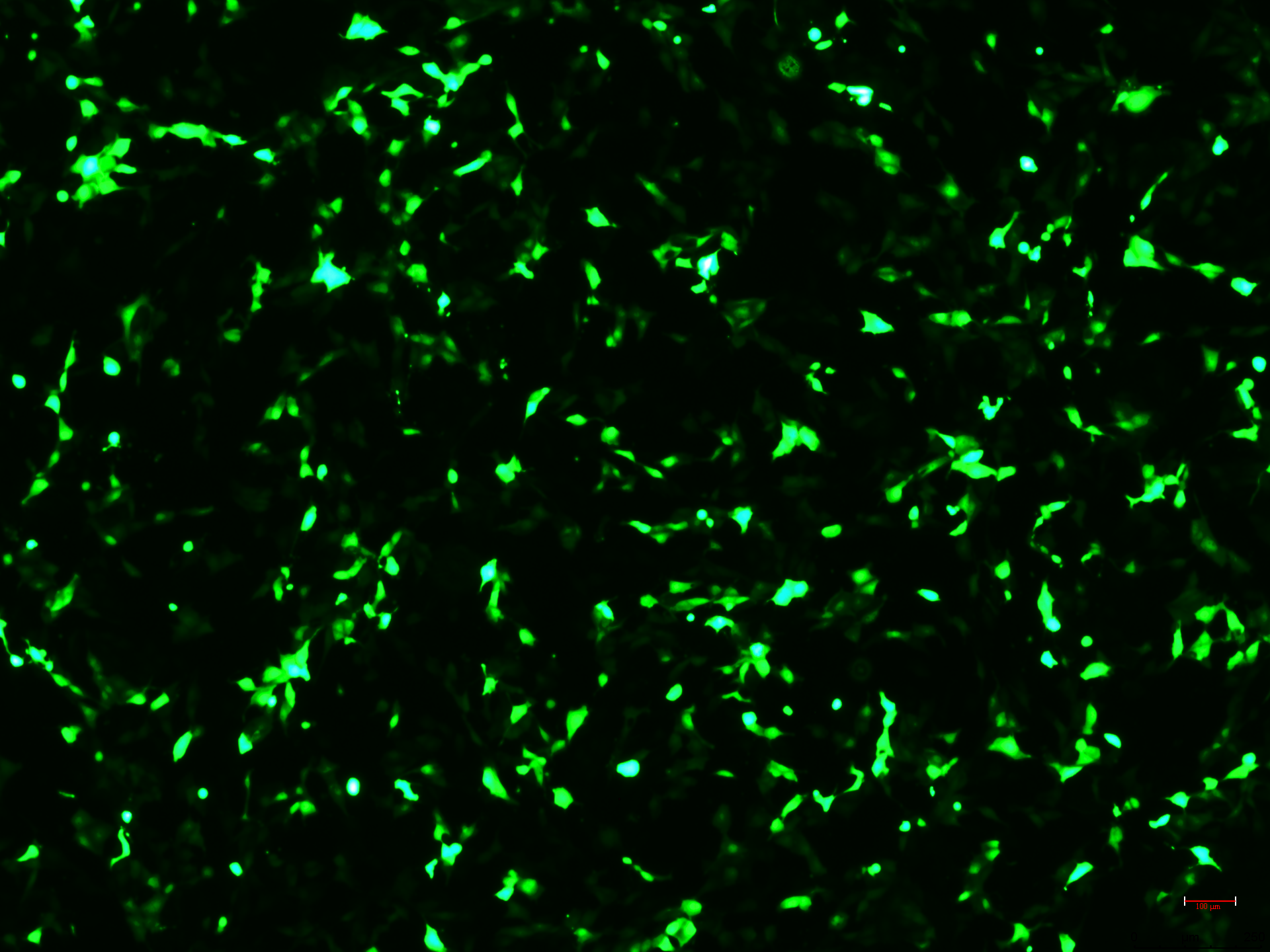

Supplement: Supplementary file 1 [file vetsci-09-00600-s001.zip › vetsci-1934507-supplementary/original source points/Overexpression/GFP/CXCL11-OE (1).tif]

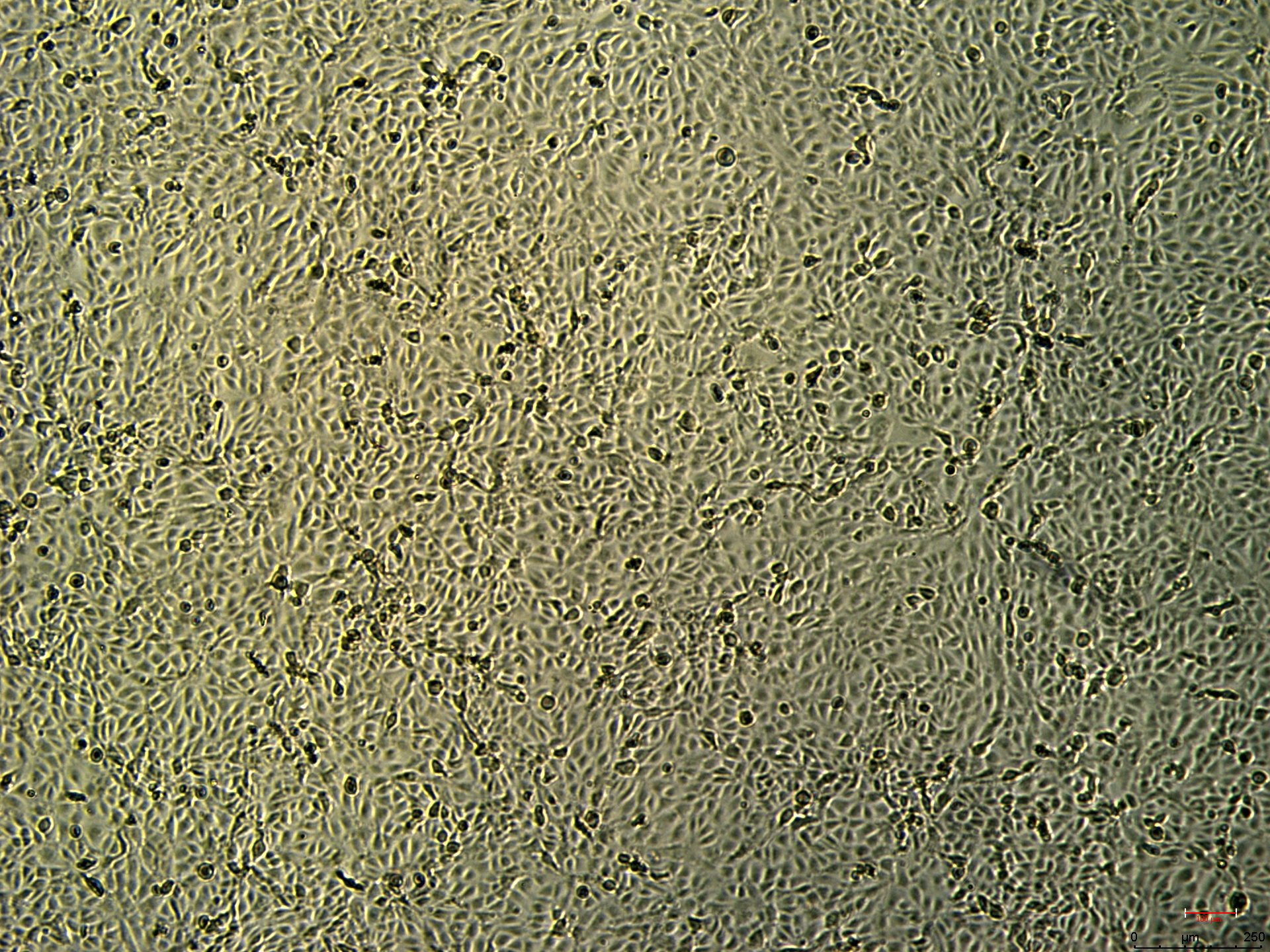

Supplement: Supplementary file 1 [file vetsci-09-00600-s001.zip › vetsci-1934507-supplementary/original source points/Overexpression/GFP/CXCL11-OE (2).tif]

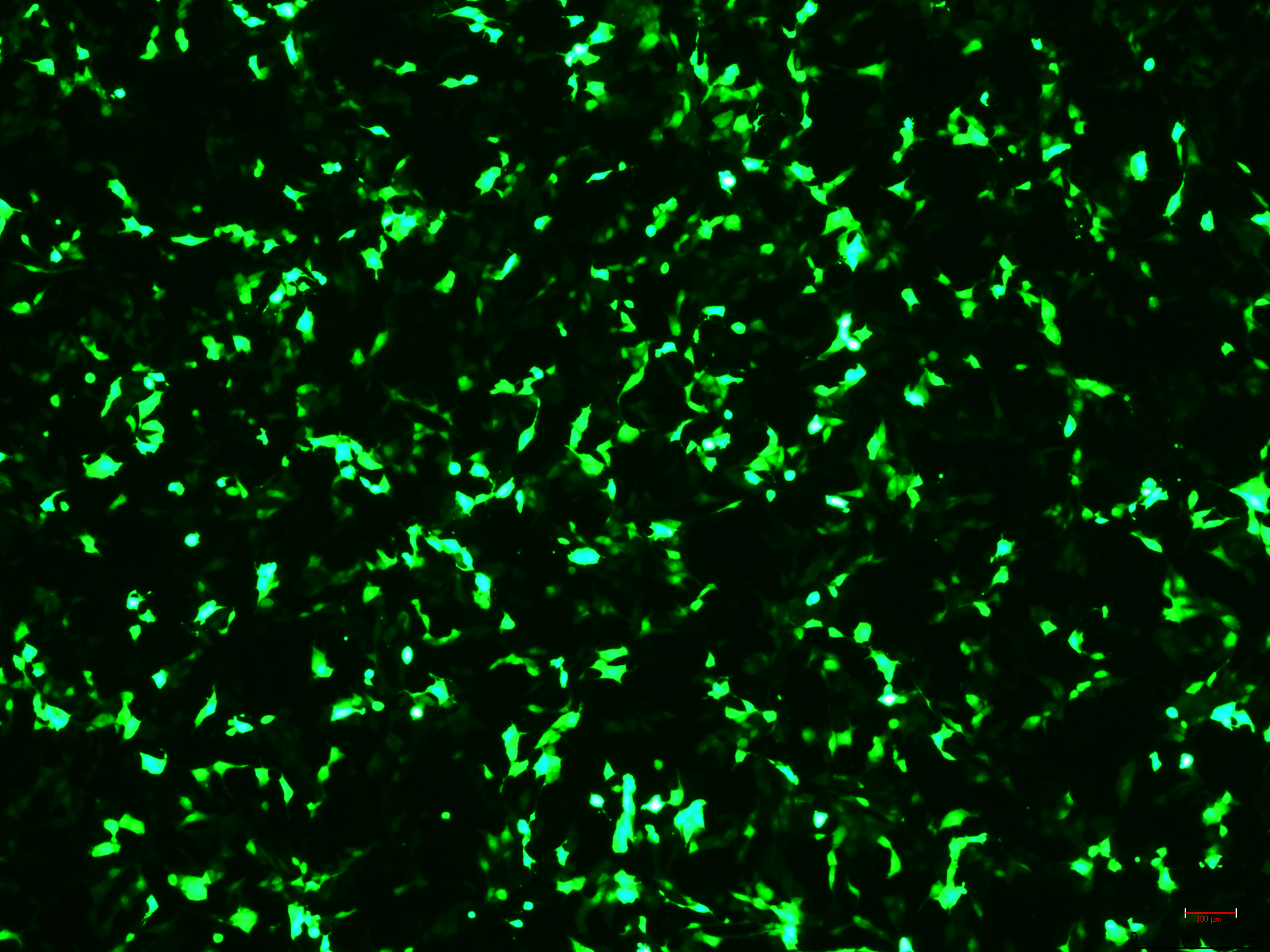

Supplement: Supplementary file 1 [file vetsci-09-00600-s001.zip › vetsci-1934507-supplementary/original source points/Overexpression/GFP/NC-OE (1).tif]

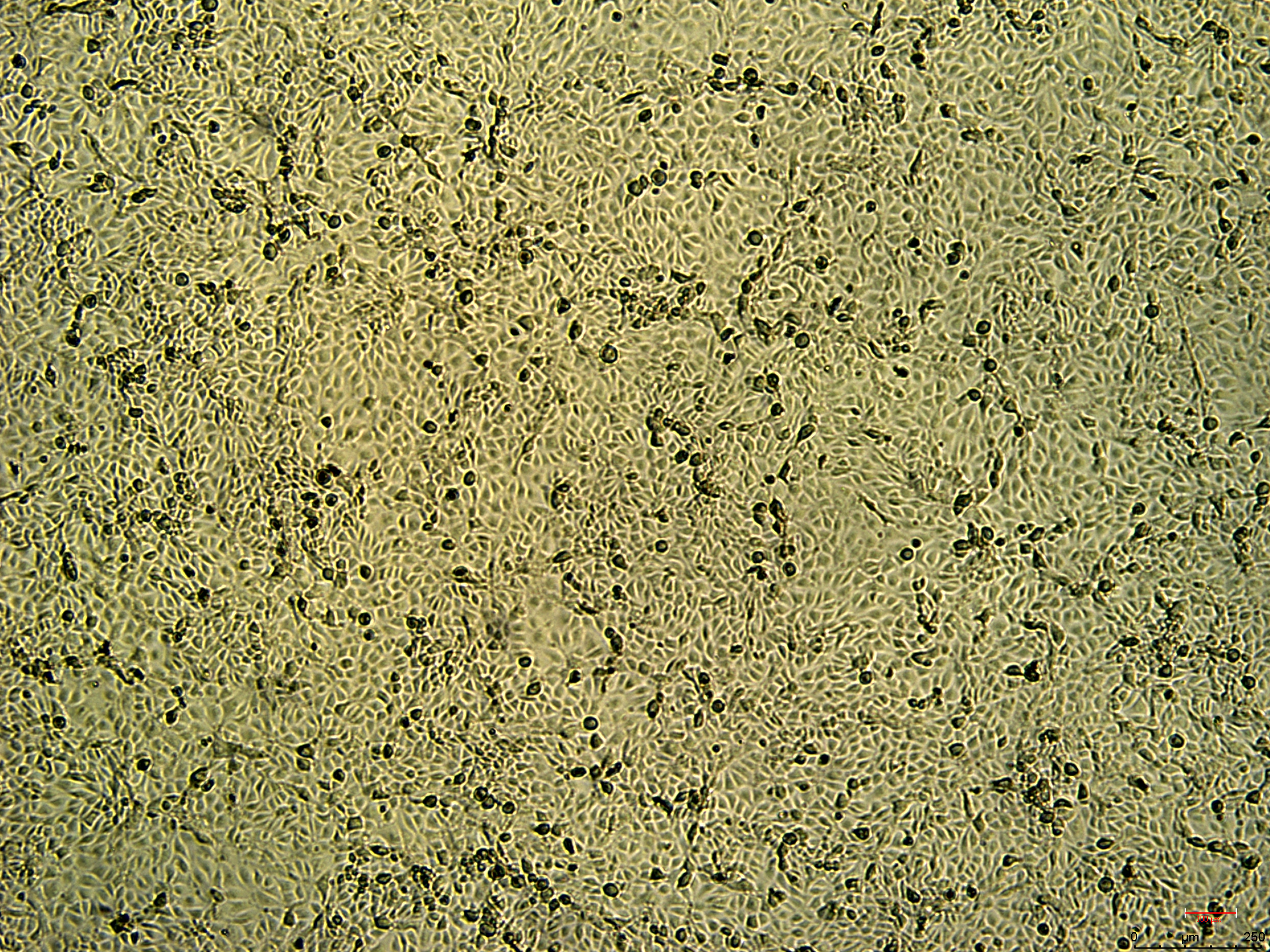

Supplement: Supplementary file 1 [file vetsci-09-00600-s001.zip › vetsci-1934507-supplementary/original source points/Overexpression/GFP/NC-OE (2).tif]

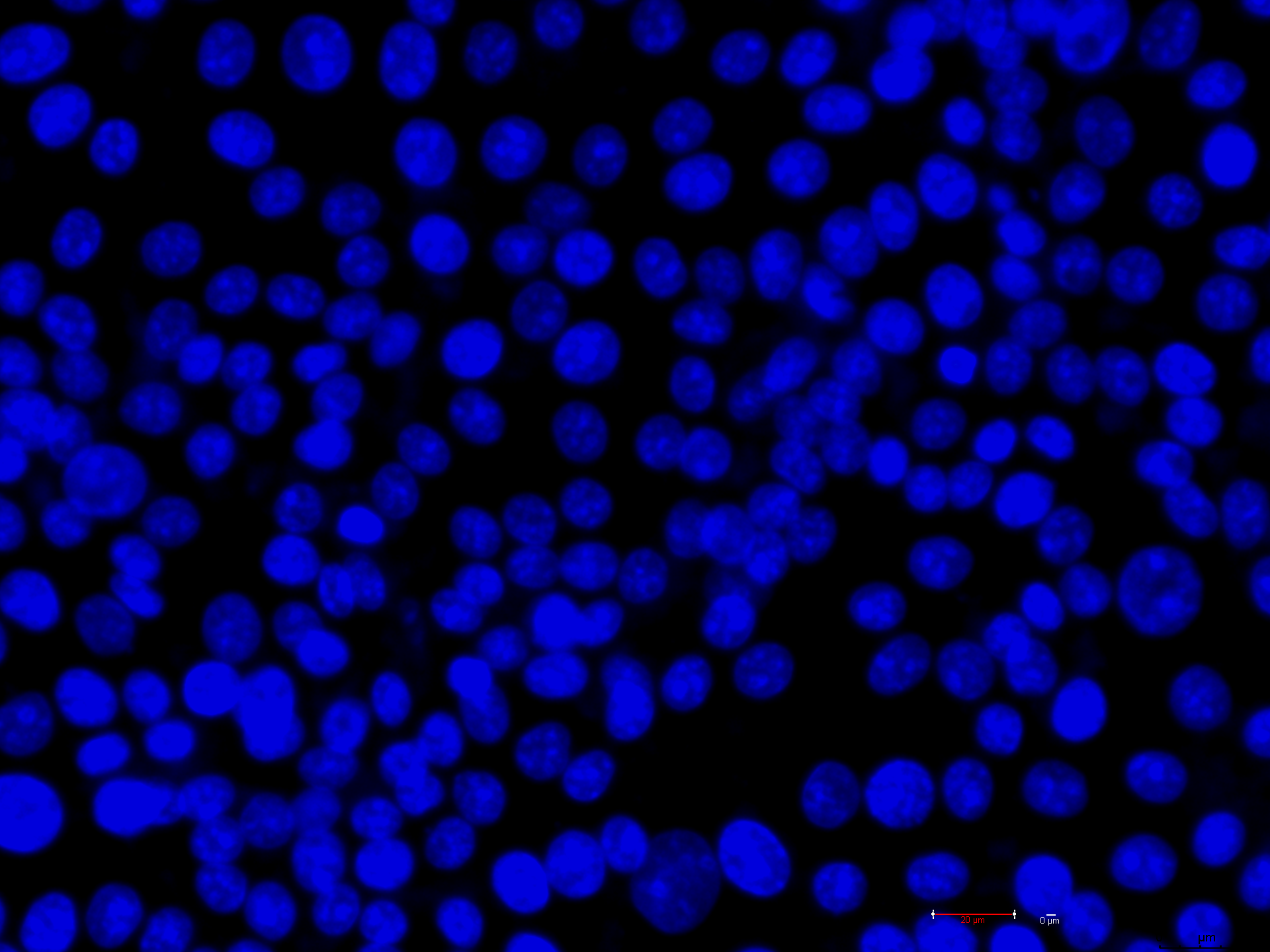

Supplement: Supplementary file 1 [file vetsci-09-00600-s001.zip › vetsci-1934507-supplementary/original source points/Overexpression/Immunofluorescence assay (IFA)/CXCL11-OE (DAPI).tif]

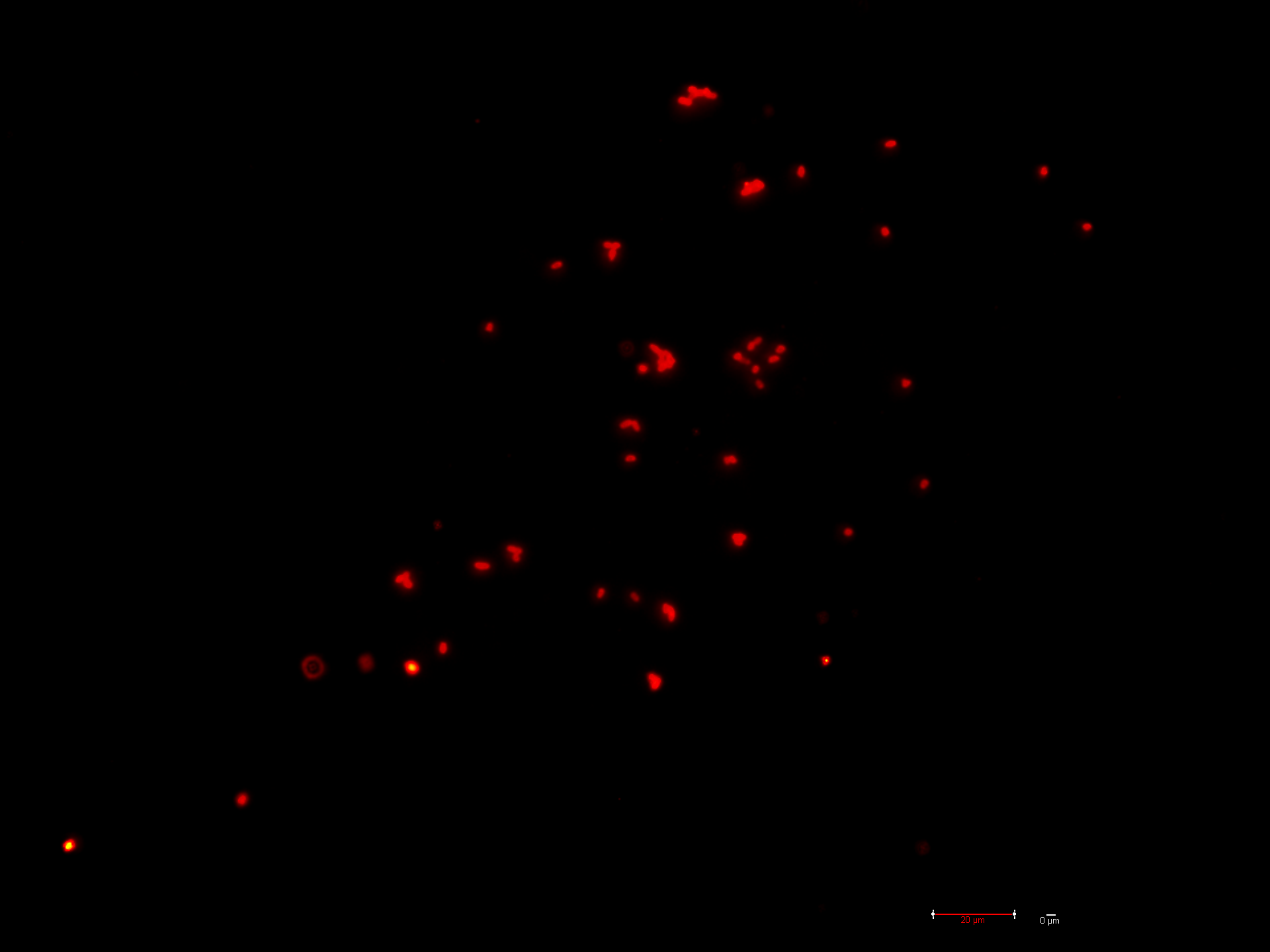

Supplement: Supplementary file 1 [file vetsci-09-00600-s001.zip › vetsci-1934507-supplementary/original source points/Overexpression/Immunofluorescence assay (IFA)/CXCL11-OE (E. coli F18).tif]

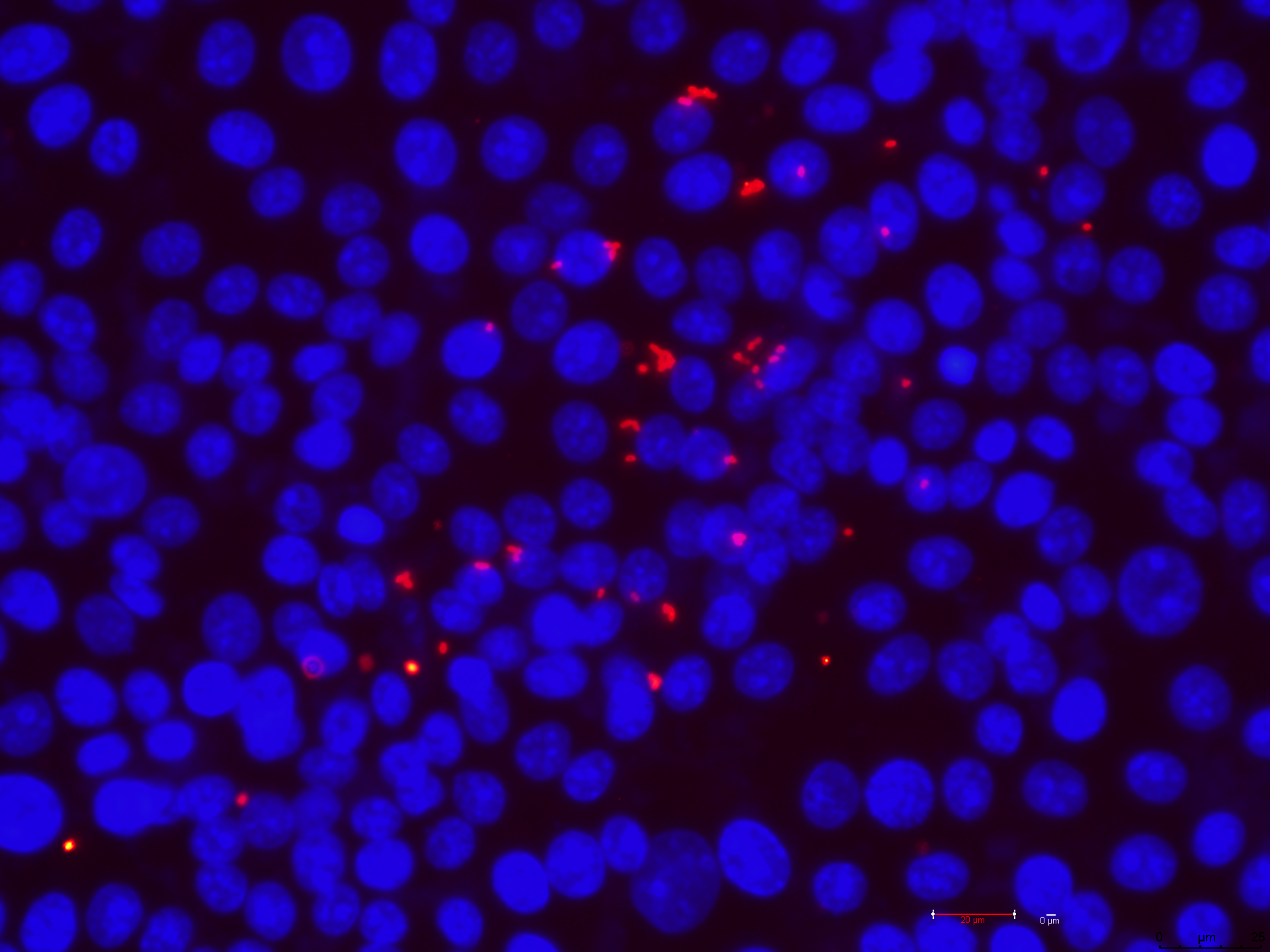

Supplement: Supplementary file 1 [file vetsci-09-00600-s001.zip › vetsci-1934507-supplementary/original source points/Overexpression/Immunofluorescence assay (IFA)/CXCL11-OE (Merge).tif]

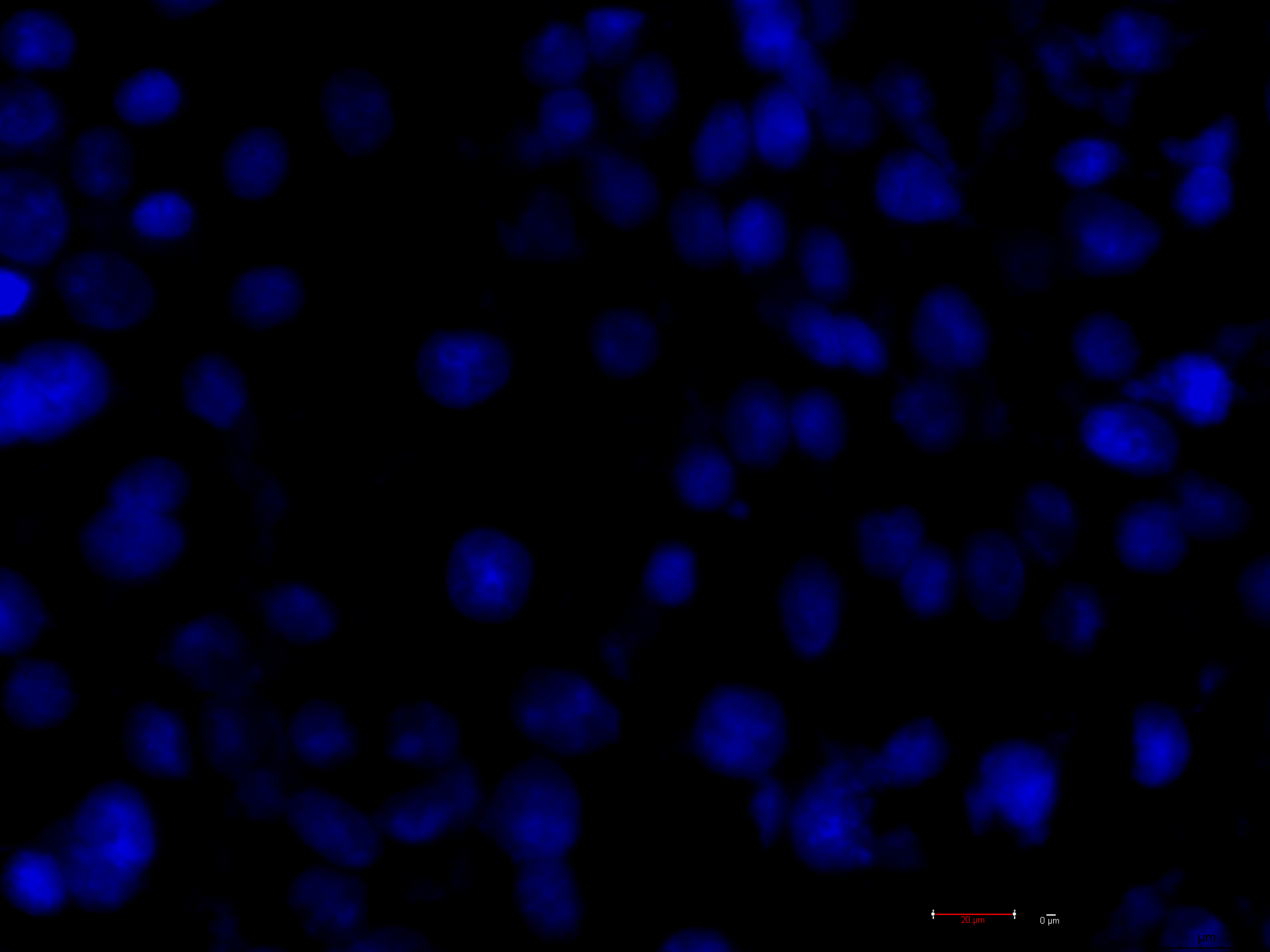

Supplement: Supplementary file 1 [file vetsci-09-00600-s001.zip › vetsci-1934507-supplementary/original source points/Overexpression/Immunofluorescence assay (IFA)/NC-OE (DAPI).tif]

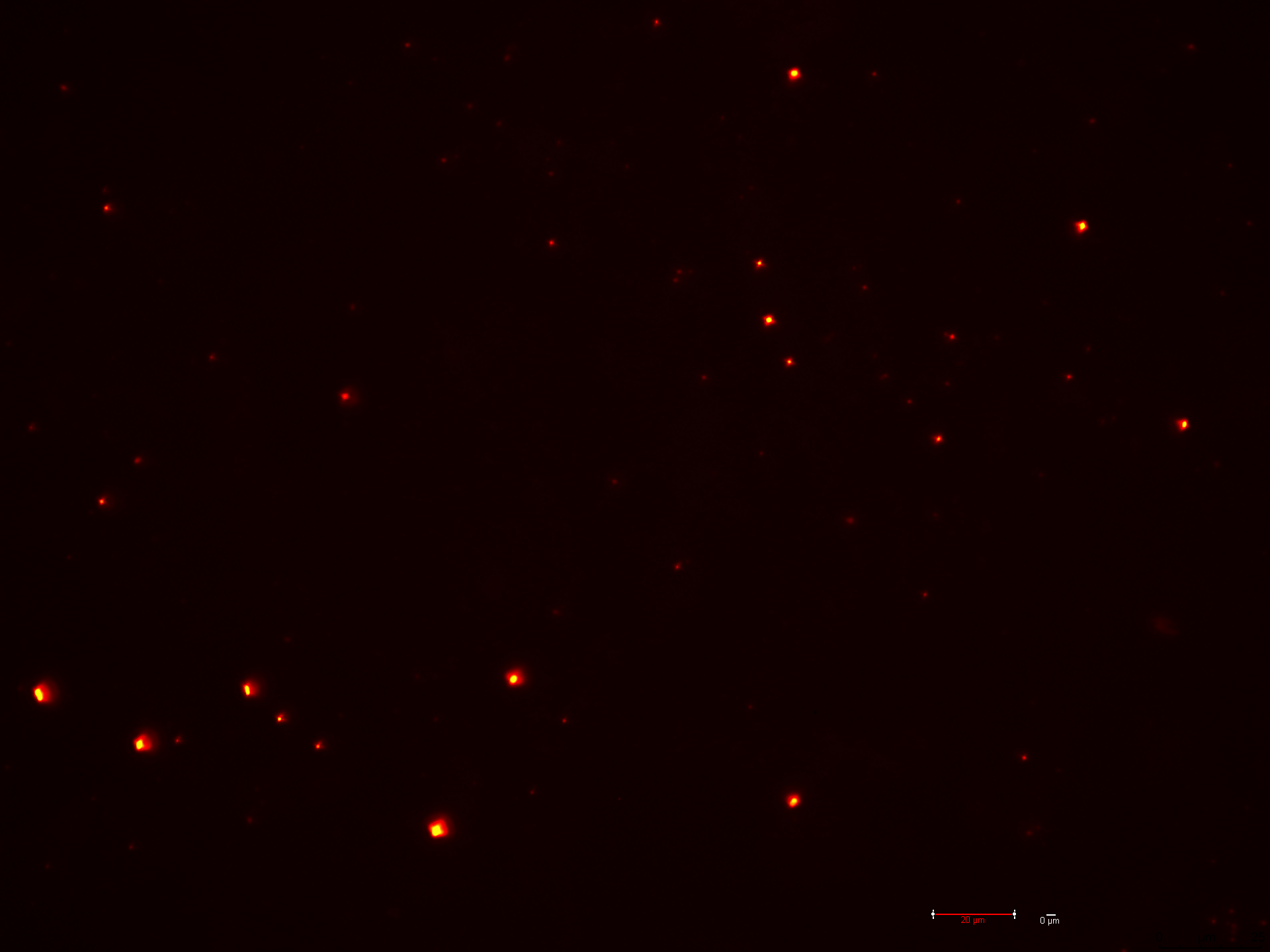

Supplement: Supplementary file 1 [file vetsci-09-00600-s001.zip › vetsci-1934507-supplementary/original source points/Overexpression/Immunofluorescence assay (IFA)/NC-OE (E. coli F18).tif]

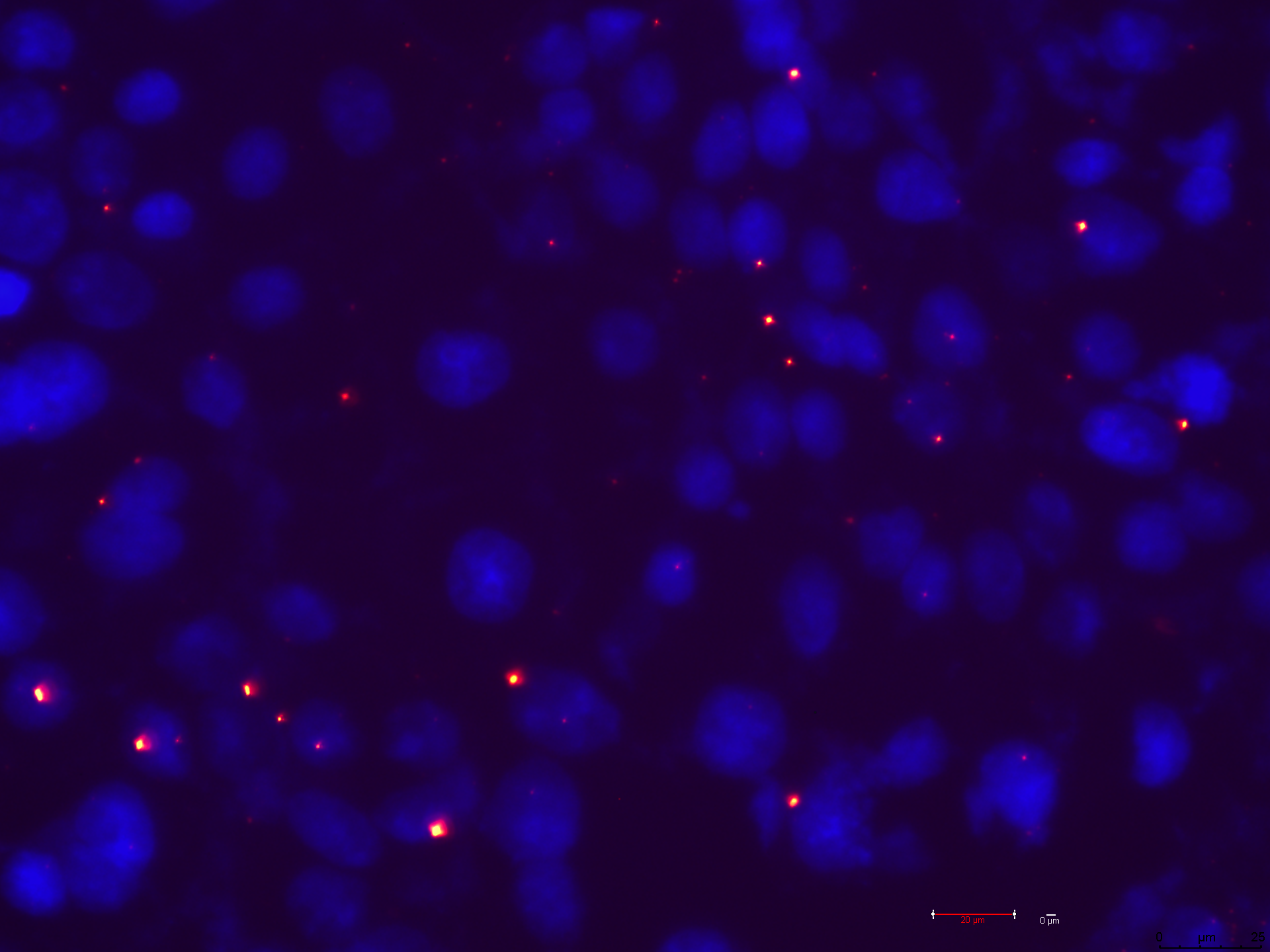

Supplement: Supplementary file 1 [file vetsci-09-00600-s001.zip › vetsci-1934507-supplementary/original source points/Overexpression/Immunofluorescence assay (IFA)/NC-OE (Merge).tif]

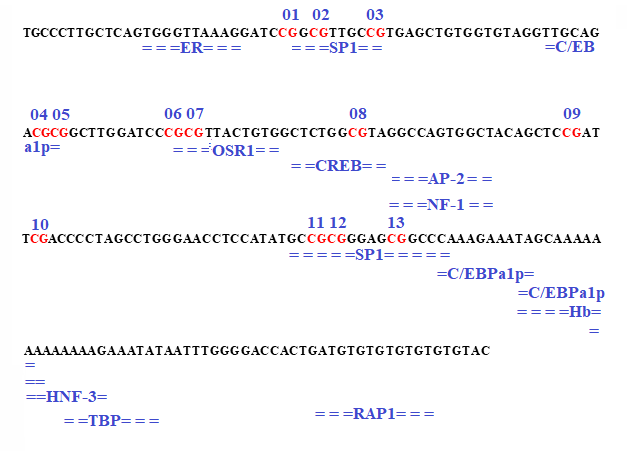

Supplement: Supplementary file 1 [file vetsci-09-00600-s001.zip › vetsci-1934507-supplementary/original source points/Transcription factor (TF)/TF.tif]

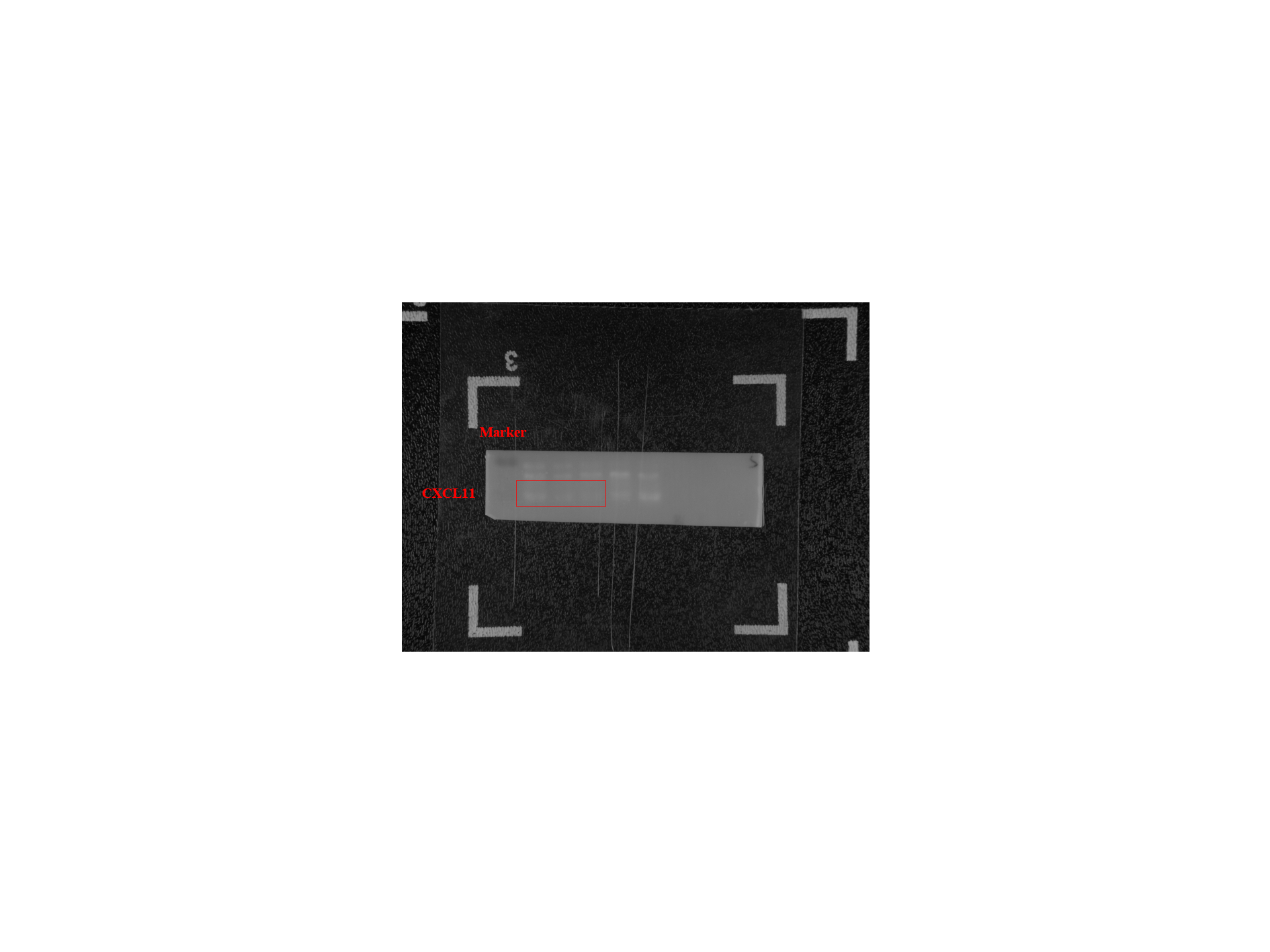

Supplement: Supplementary file 1 [file vetsci-09-00600-s001.zip › vetsci-1934507-supplementary/original source points/Westen blot/Figure S1B/Figure S1B CXCL11(1).tif]

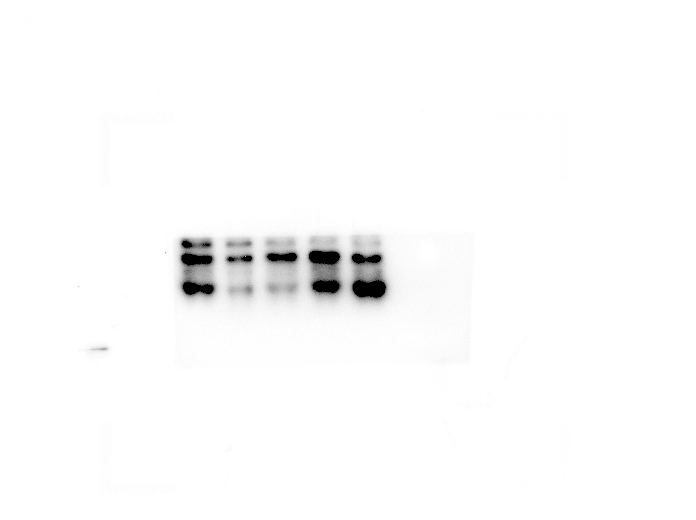

Supplement: Supplementary file 1 [file vetsci-09-00600-s001.zip › vetsci-1934507-supplementary/original source points/Westen blot/Figure S1B/Figure S1B CXCL11(2).tif]

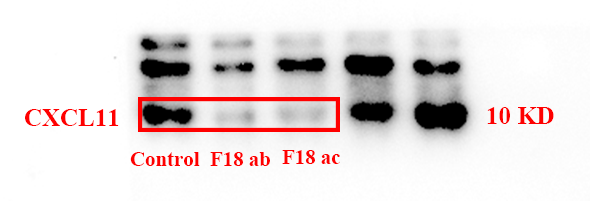

Supplement: Supplementary file 1 [file vetsci-09-00600-s001.zip › vetsci-1934507-supplementary/original source points/Westen blot/Figure S1B/Figure S1B CXCL11(3).tif]

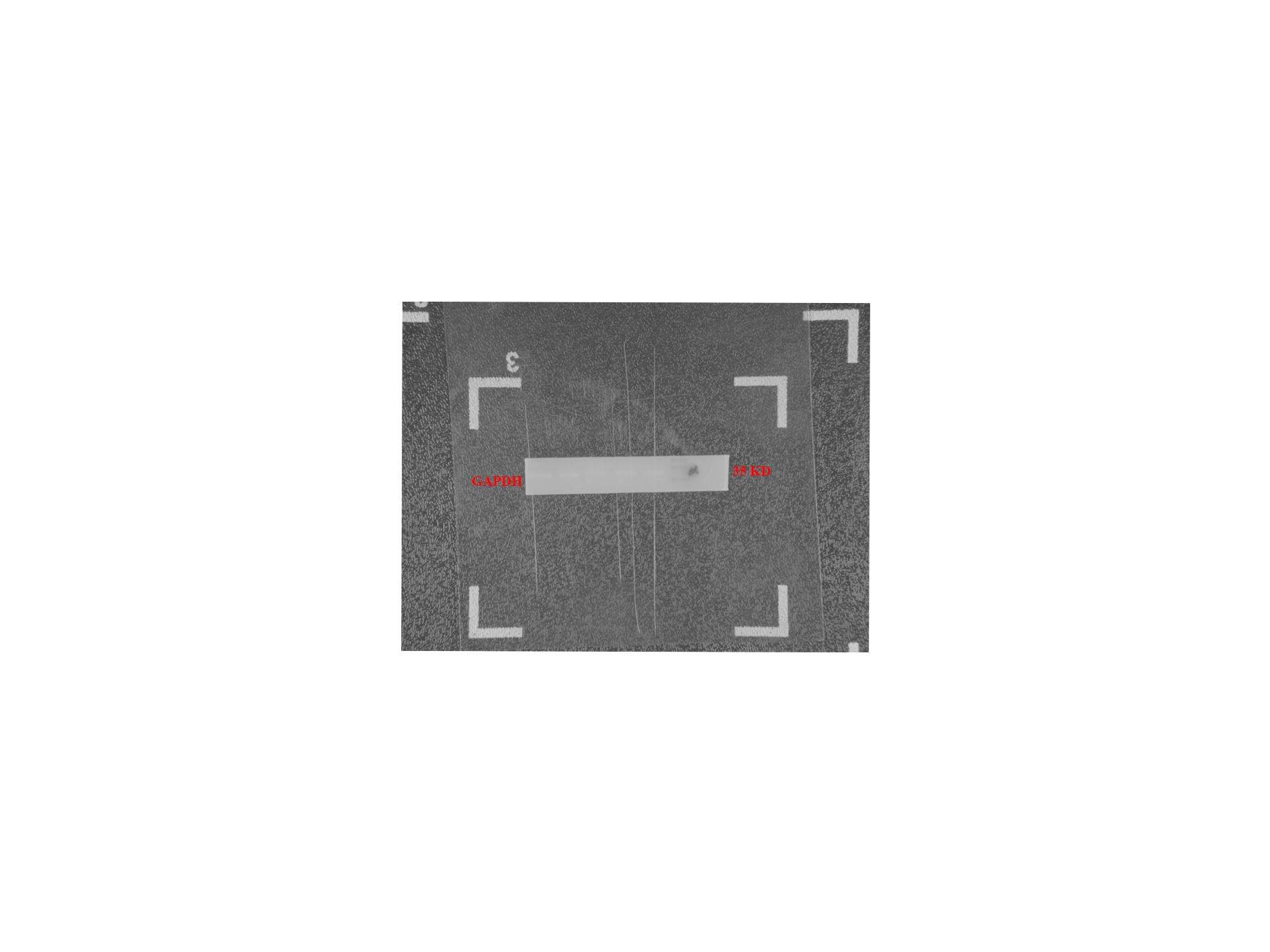

Supplement: Supplementary file 1 [file vetsci-09-00600-s001.zip › vetsci-1934507-supplementary/original source points/Westen blot/Figure S1B/Figure S1B GAPDH(1).tif.tif]

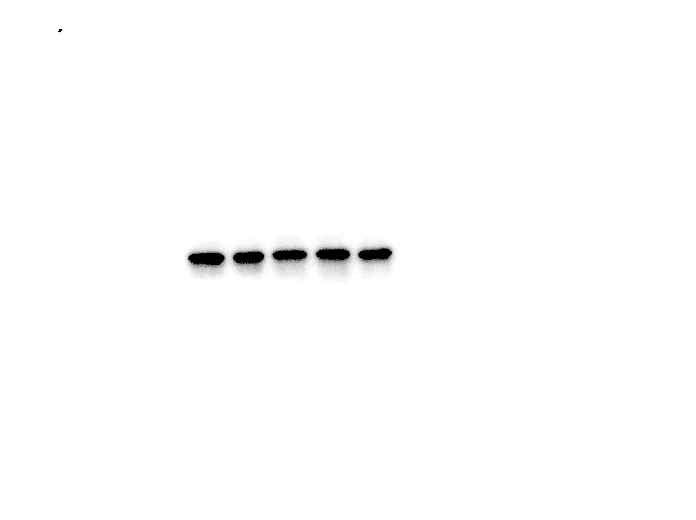

Supplement: Supplementary file 1 [file vetsci-09-00600-s001.zip › vetsci-1934507-supplementary/original source points/Westen blot/Figure S1B/Figure S1B GAPDH(2).tif]

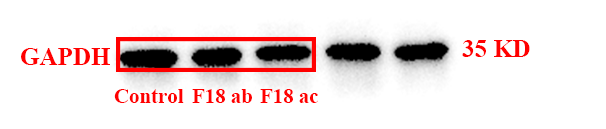

Supplement: Supplementary file 1 [file vetsci-09-00600-s001.zip › vetsci-1934507-supplementary/original source points/Westen blot/Figure S1B/Figure S1B GAPDH(3).tif.tif]

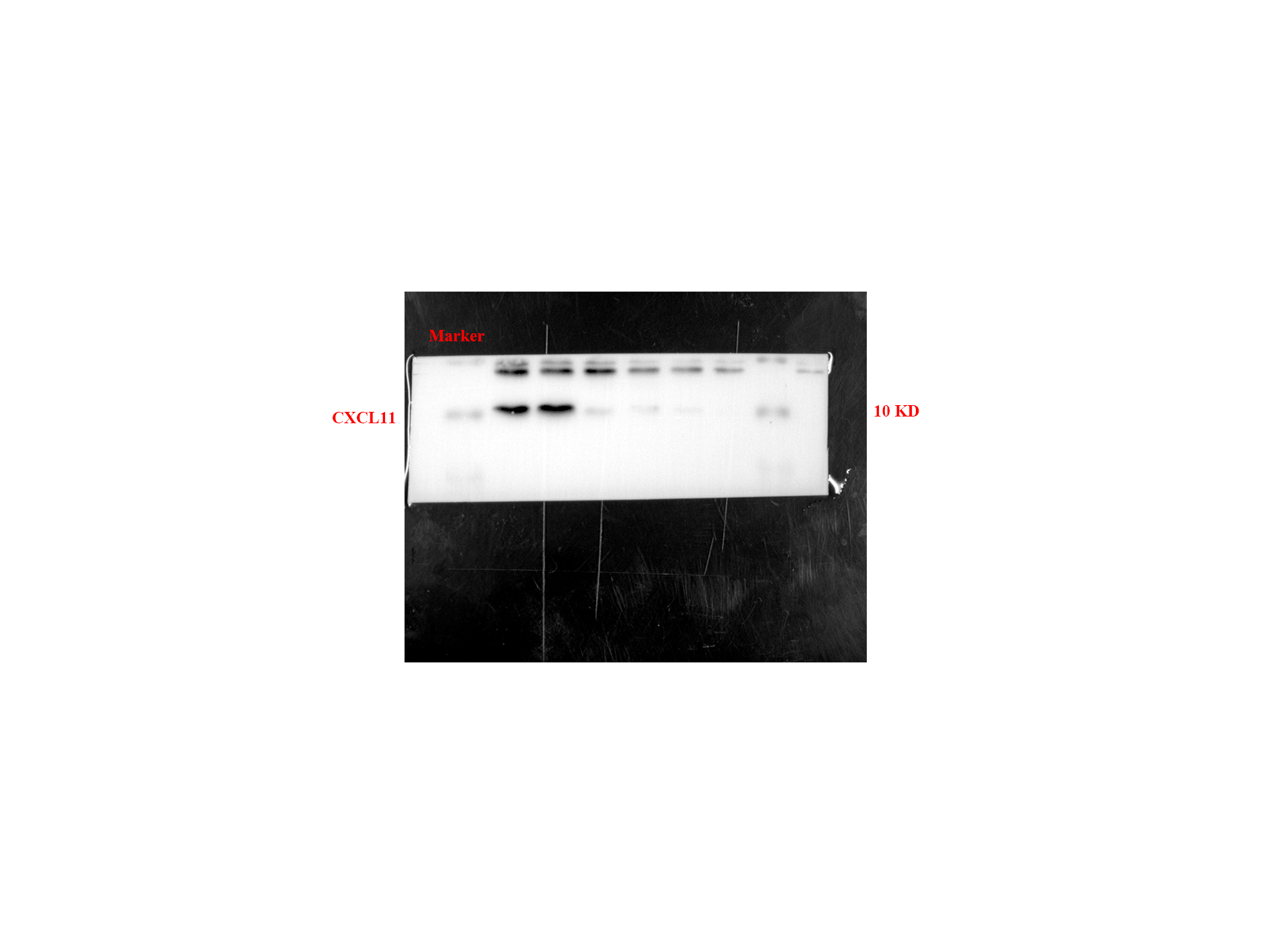

Supplement: Supplementary file 1 [file vetsci-09-00600-s001.zip › vetsci-1934507-supplementary/original source points/Westen blot/Figure S2C/Figure S2C CXCL11(1).tif]

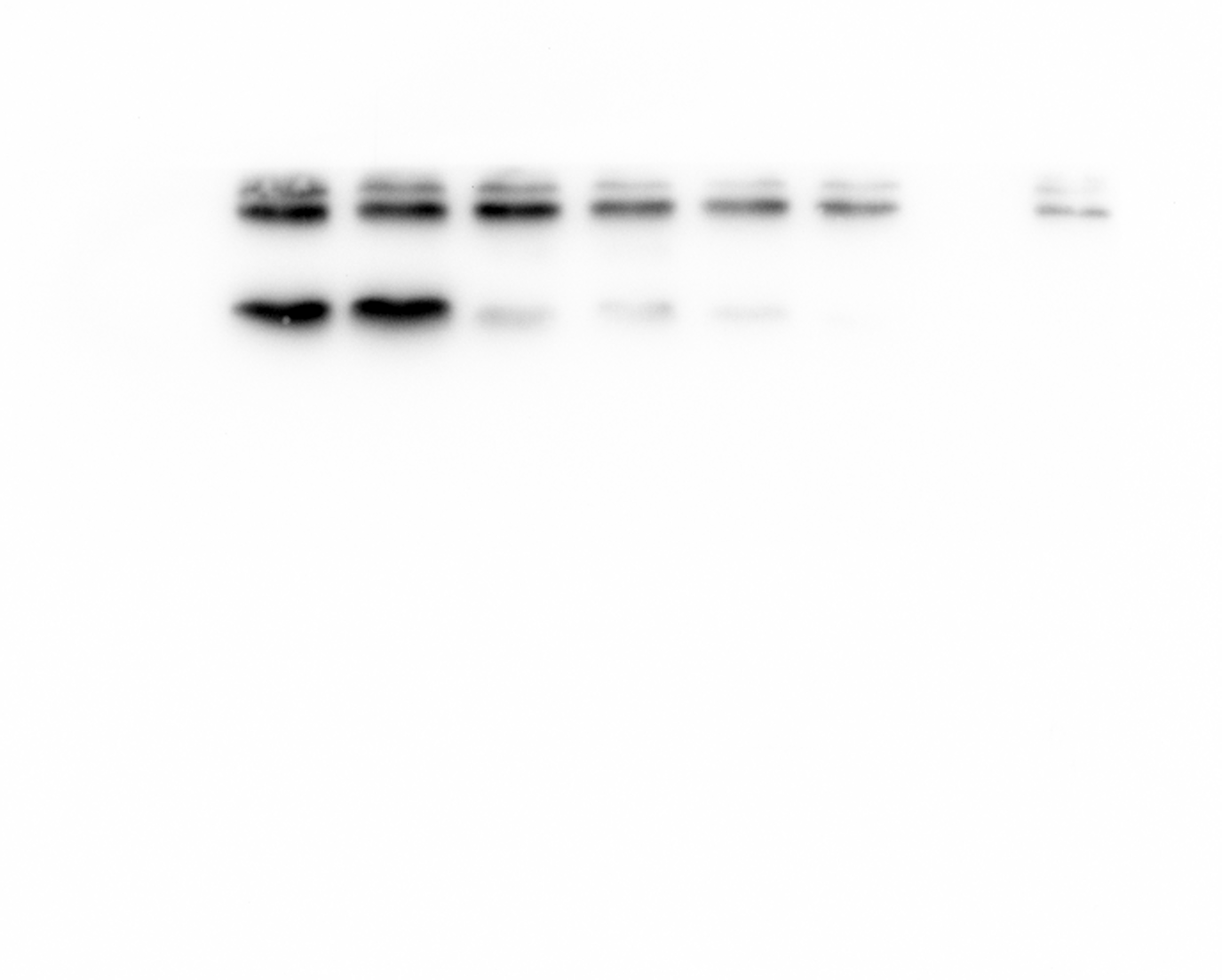

Supplement: Supplementary file 1 [file vetsci-09-00600-s001.zip › vetsci-1934507-supplementary/original source points/Westen blot/Figure S2C/Figure S2C CXCL11(2).Tif]

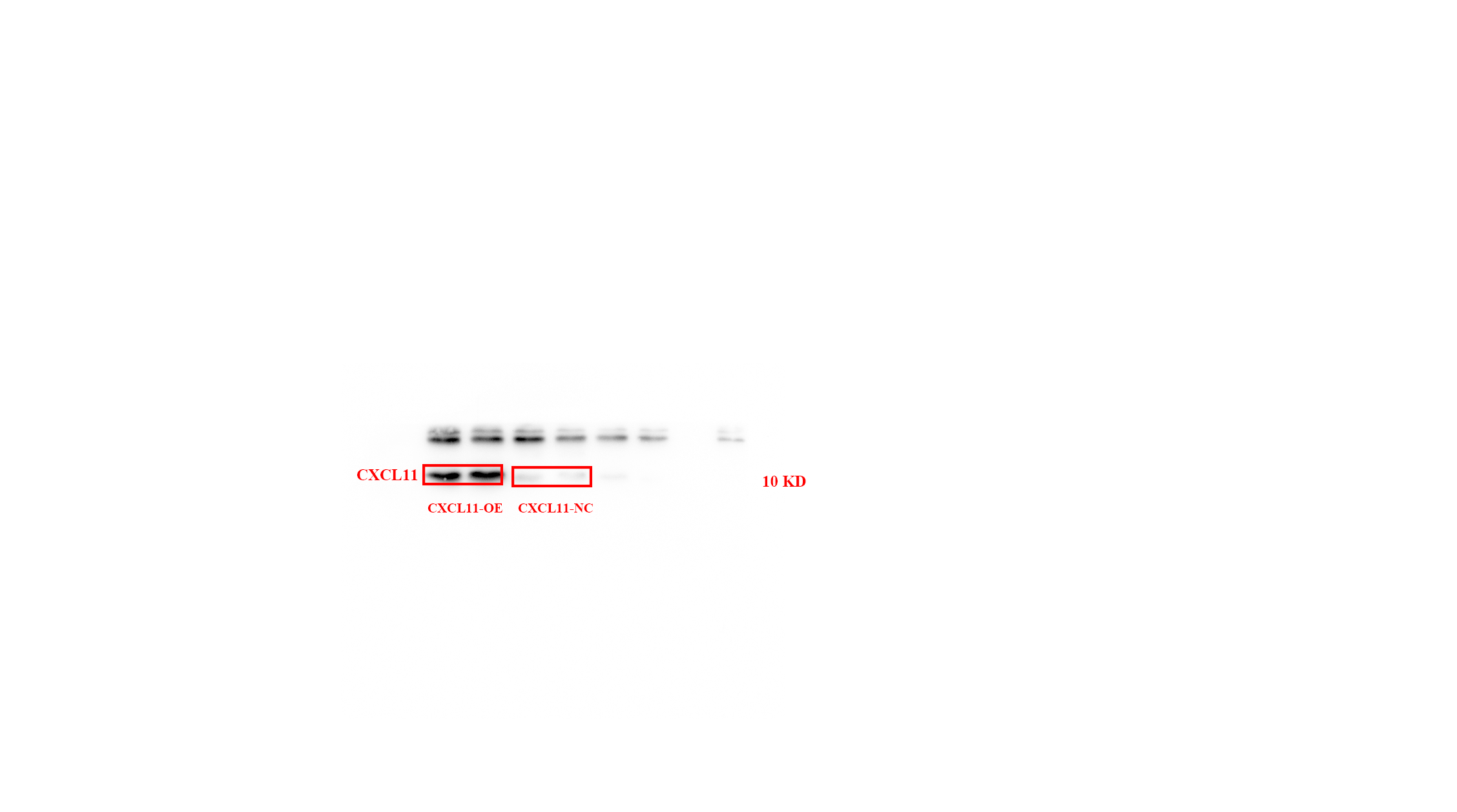

Supplement: Supplementary file 1 [file vetsci-09-00600-s001.zip › vetsci-1934507-supplementary/original source points/Westen blot/Figure S2C/Figure S2C CXCL11(3).tif]

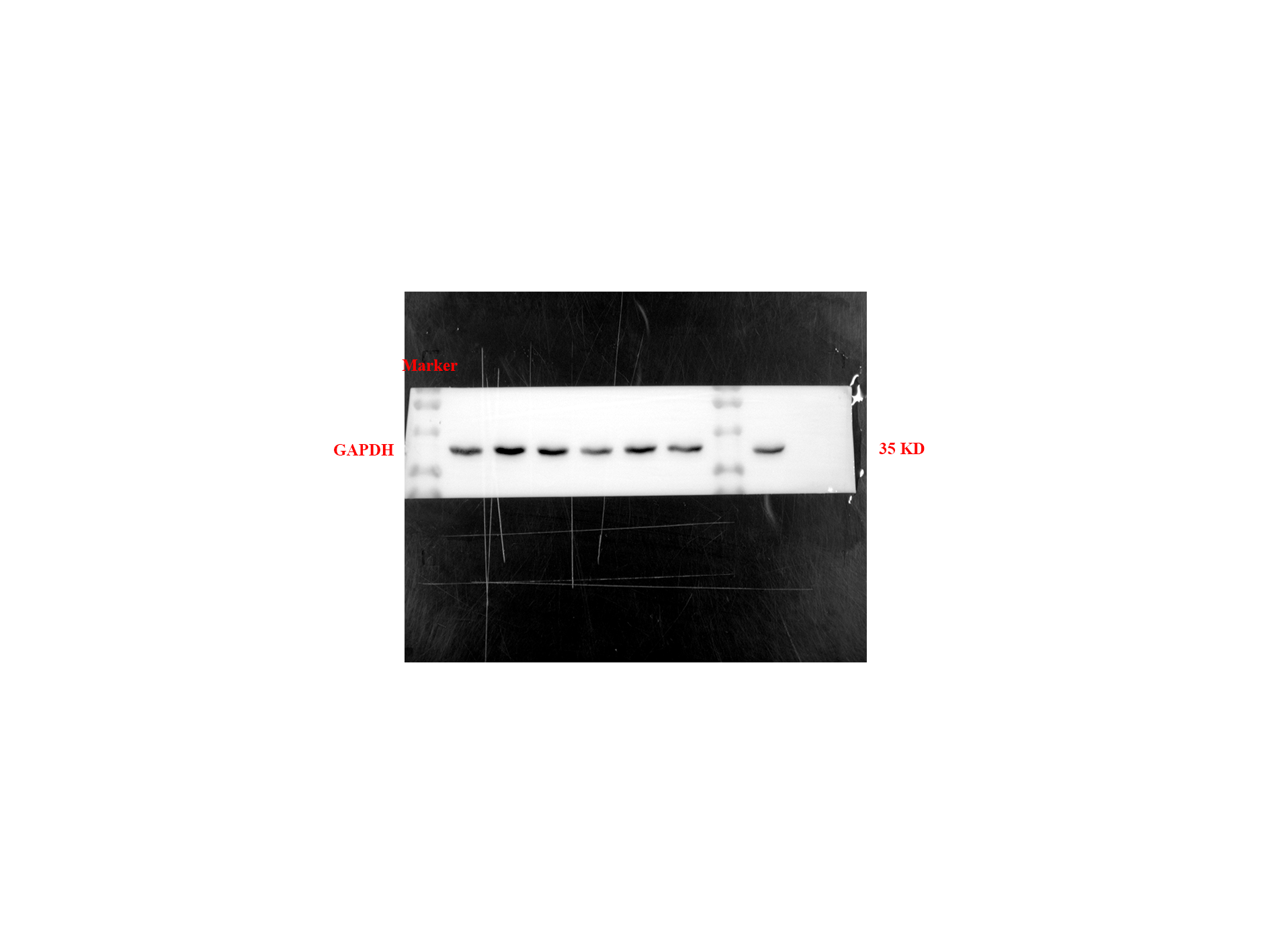

Supplement: Supplementary file 1 [file vetsci-09-00600-s001.zip › vetsci-1934507-supplementary/original source points/Westen blot/Figure S2C/Figure S2C GAPDH(1).tif]

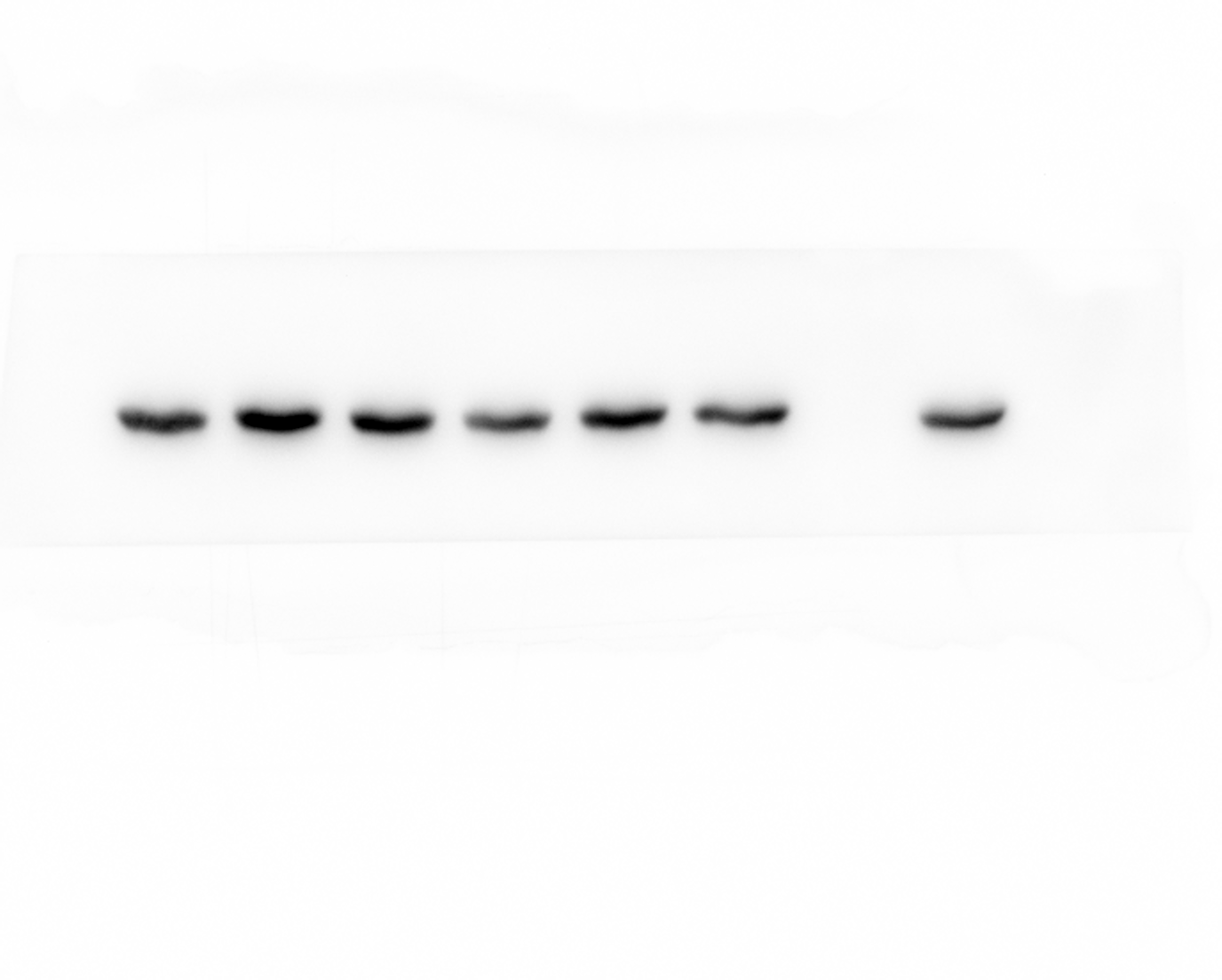

Supplement: Supplementary file 1 [file vetsci-09-00600-s001.zip › vetsci-1934507-supplementary/original source points/Westen blot/Figure S2C/Figure S2C GAPDH(2).Tif]

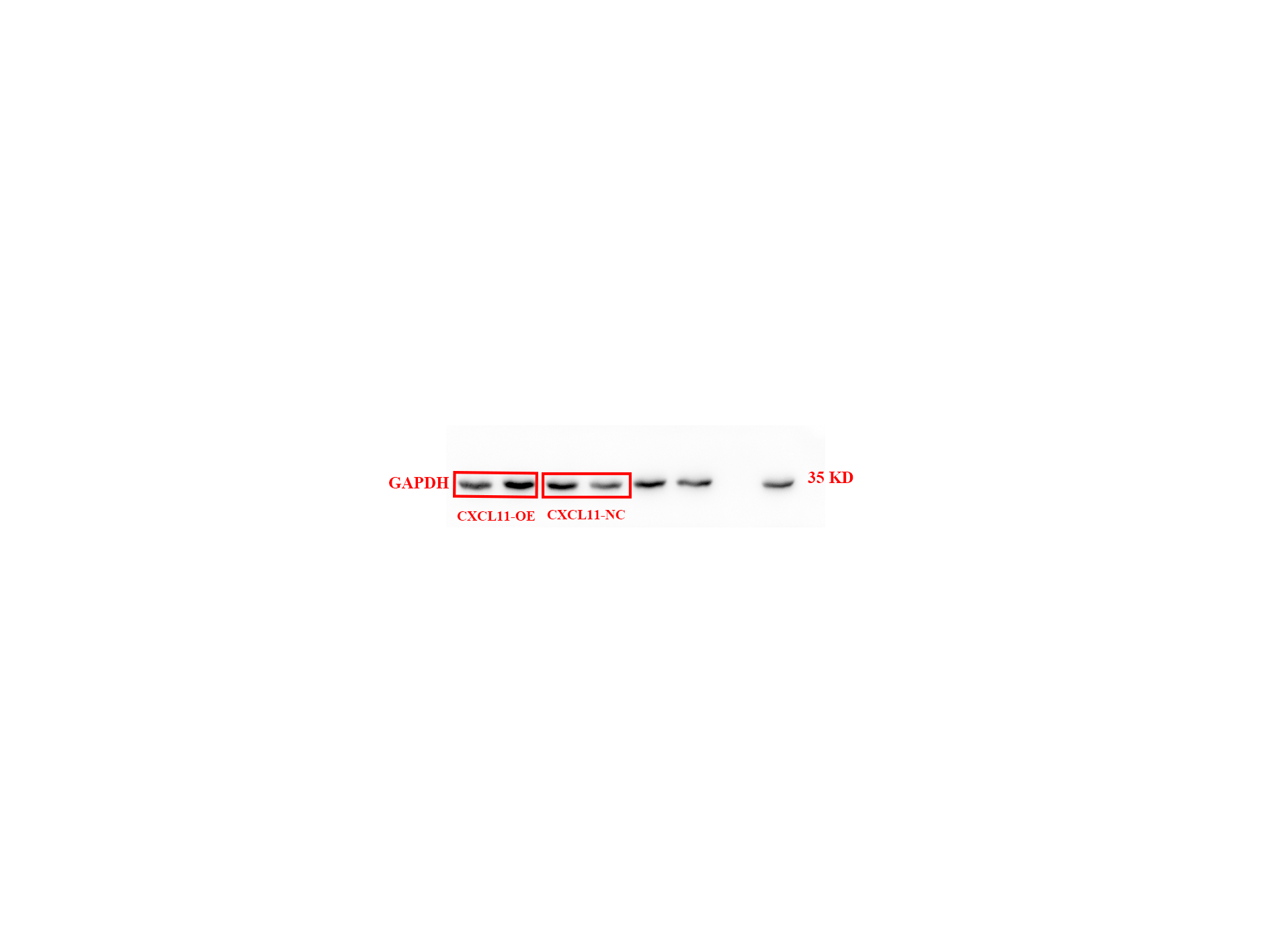

Supplement: Supplementary file 1 [file vetsci-09-00600-s001.zip › vetsci-1934507-supplementary/original source points/Westen blot/Figure S2C/Figure S2C GAPDH.tif]
